# Patch-seq analysis reveals transcriptomic correlates of neuronal firing rate adaptation across subtypes and species

**DOI:** 10.1016/j.isci.2026.117712

**Published:** 2026-10-08

**Authors:** John Hongyu Meng, Yijie Kang, Alan Lai, Michael Feyerabend, Wataru Inoue, Julio Martinez-Trujillo, Bernardo Rudy, Xiao-Jing Wang

**Affiliations:** 1 Center for Neural Science, New York University, New York, NY 10003, USA; 2 Department of Physiology and Pharmacology, Western University, London, ON N6A 3K7, Canada; 3 Robarts Research Institute, Western University, London, ON N6A 3K7, Canada; 4 Schulich School of Medicine and Dentistry, Western University, London, ON N6A 3K7, Canada; 5 Neuroscience Institute, NYU Grossman School of Medicine, New York, NY 10016, USA; 6 Department of Neuroscience and Physiology, NYU Grossman School of Medicine, New York, NY 10016, USA; 7 Department of Anesthesiology, Perioperative Care and Pain Medicine, NYU Grossman School of Medicine, New York, NY 10016, USA

**Keywords:** Patch-seq, spike-rate adaptation, transcriptomics, electrophysiology, ion channels, neuronal excitability, interneurons, calcium channels

## Abstract

Can the transcriptomic profile of a neuron predict its physiological properties? Using a Patch-seq dataset of the primary visual cortex, we addressed this question by focusing on spike rate adaptation (SRA), a well-known phenomenon that depends on small conductance calcium (Ca)-dependent potassium (SK) channels. We first show that in parvalbumin-expressing (PV) and somatostatin-expressing (SST) interneurons, expression levels of genes encoding the ion channels underlying action potential generation are correlated with the half-width (HW) of spikes. Surprisingly, the SK encoding genes are not correlated with the degree of SRA (dAdap). Instead, genes that encode proteins upstream from the SK current, as well as the low-voltage-activated calcium channel, are correlated with dAdap. This general principle is supported by datasets from the mouse primary motor cortex and multiple macaque brain regions. Finally, we generate testable predictions by constructing a minimal computational model.

## Highlights

• Spike adaptation differences in INs are not predicted by SK channel gene expression
• Upstream SK activation genes and LVA Ca channel genes predict adaptation differences
• The mechanistic principle of adaptation generalizes across regions and species
• The model generates testable predictions for adaptation mechanisms

## Graphical abstract

**Figure:**
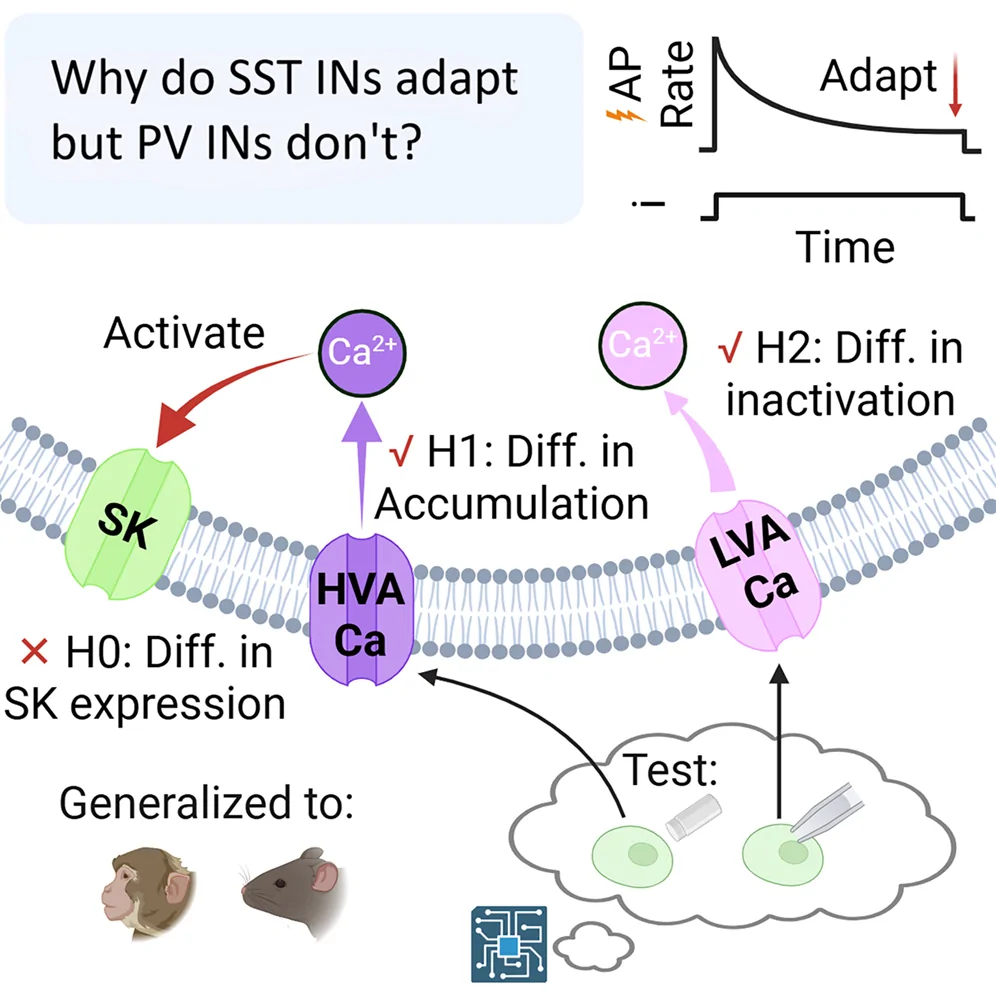

## Teaser

Neuroscience; Transcriptomics; Machine learning

## Introduction

With the advances in the brain connectome and the simultaneous recording from multiple brain areas in behaving animals, studies of the large-scale multiregional brain have come to the fore.^1^ To understand brain-wide dynamics, it is necessary to consider differences in the electrophysiological features of single neurons in different areas. However, neuron electrophysiological studies such as patch-clamp measurements are costly; at the present time there is a dearth of systematic comparisons of electrophysiological characteristics across many brain regions. On the other hand, single-cell transcriptomic analysis has become common and offers a novel approach to quantification of brain diversity and heterogeneity.^2^^,^^3^^,^^4^ So far, this tool has been mostly deployed for cell-type classification. Can a transcriptomic profile predict the physiological properties of different cell types? The answer is not obvious, as there are multiple intermediate steps from mRNAs to proteins to physiological functions of receptors and ion channels. In this work, we tackled this challenging question by focusing on single-neuron spike rate adaptation (SRA), a salient characteristic of firing patterns in distinct subtypes of neurons.

The development of patch-seq marks a significant advancement in correlating transcriptomic data with single-cell electrophysiological features.^5^ Unlike quantitative real-time PCR (RT-PCR), which is limited to studying specific genes with high accuracy,^6^ and single-cell RNA sequencing (scRNA-seq), which provides a genome-wide expression profile across diverse neuron types and species,^4^ patch-seq combines scRNA-seq with patch-clamp recordings. This method simultaneously captures RNA sequencing, electrophysiological, and morphological data from the same neuron, providing data at an unprecedented resolution. This offers a novel approach to understanding neural computation at the molecular level. Previous work has performed unsupervised analysis of patch-seq datasets to generate a list of genes that correlate with electrophysiological or morphological properties.^7^ However, this approach typically limits the analysis to correlations between individual genes and features of interest, making it less likely to capture the complex interactions between various genes and provide a causal explanation of the underlying mechanisms.

To accurately predict electrophysiological features from transcriptomic data without overfitting to region-specific genes, we adapt a comprehensive approach that considers the broader network of gene interactions in addition to gene-feature correlations. We started with one important feature of single neurons: SRA.^8^ This feature was first discovered in,^9^ describing how neurons modulate their output to the same input. It is important in various cognitive functions that have been validated by experimental and theoretical works alike: sensory processing from vision,^10^ audition,^11^ and olfaction^12^; working memory in language processing^13^; perceptual bistability^14^ and multistable perceptions^15^; theta sweep in place cells^16^; precision in spike-timing^17^; and processing of temporally dispersed information.^18^ Interestingly, different cell types show large differences in the SRA.^19^ Parvalbumin (PV) interneurons (IN), recognized by their signature narrow action potential (AP) waveform, show no or little SRA. Somatostatin (SST) INs, which share the same origins from medial ganglionic eminence (MGE) as PV INs,^20^ in general they have a broader AP waveform and a stronger adaptation compared to PV. PV and SST are widely distributed in the layers and regions of the cortex and account for about 70% of the total INs of the whole cortex,^19^ although some variability between cortical areas has been reported.^21^ These features make them ideal first targets for studying the mechanisms behind the SRA.

In prior studies, the mechanisms underlying firing-rate adaptation have been examined primarily in pyramidal cells.^22^ Typically, an AP activates high-voltage-activated (HVA) calcium (Cav) channels, particularly L-type. The resulting *Ca*^2+^ influx through these L-type Cav channels further activates small-conductance calcium-activated potassium (SK) channels, which generate an outward after hyperpolarization potential (AHP) that slows the regeneration of subsequent APs.^23^ However, little is known about whether this mechanism generalizes to INs. In addition to the SK pathway, other mechanisms may contribute, such as inactivation of low-voltage-activated (LVA) Cav channels, calcium buffering systems,^22^ M-type K^+^ channels, and the hyperpolarization-activated cation (H) current.^24^

Here, we utilize the open patch-seq dataset^25^ recorded from the primary visual cortex (V1), where both electrophysiological and transcriptomic data are available for PV and SST cells, to search for transcriptomic correlates of firing-rate adaptation. We first show that SRA, measured by the degree of adaptation (dAdap), is a key feature in distinguishing PV and SST cells. We further find that the AP half-width (HW) and dAdap are highly correlated at the single-cell level. Next, we seek the transcriptomic correlation of adaptation using the known transcriptomic correlation of HW as a control. We develop a method to systematically de-noise the original patch-seq data at the transcriptomic-defined subtype (T-type) level and further validate that HW differences correlate with the transcriptomic data at the T-type level. We hypothesize that the SK channel encoding gene should correlate with dAdap, but we surprisingly find that the data do not support this. Instead, the Ca influx-related mechanisms, namely HW and HVA Cav channel encoding genes, can explain the observed dAdap differences. In addition, the inactivation of LVA Cav channel explains a fraction of the observed differences. We predict other genes that are likely involved in modulating firing-rate adaptation. We further extended our analysis to a mouse M1 patch-seq dataset that includes pyramidal cells,^26^ as well as to an electrophysiological and a transcriptomic dataset^3^ covering the same three brain regions in macaque monkeys, thereby confirming the generality of our conclusions. Finally, we built a Hodgkin-Huxley-type model that reproduces the observed experimental data and generates testable pharmacological predictions.

## Results

### Spike rate adaptation and spike width are correlated in PV and SST interneurons

PV and SST INs together account for about 70% of the total INs of the whole cortex.^19^ Here, we analyze the electrophysiological features of PV and SST cells from the open patch-seq dataset from the mouse V1^25^. PV cells show little adaptation over a 1s-long square-pulse injection current, while SST cells show strong adaptation. Furthermore, PV cells show the signature narrow AP waveform, while SST cells show a wider AP waveform (Figure 1A).

**Figure 1:**
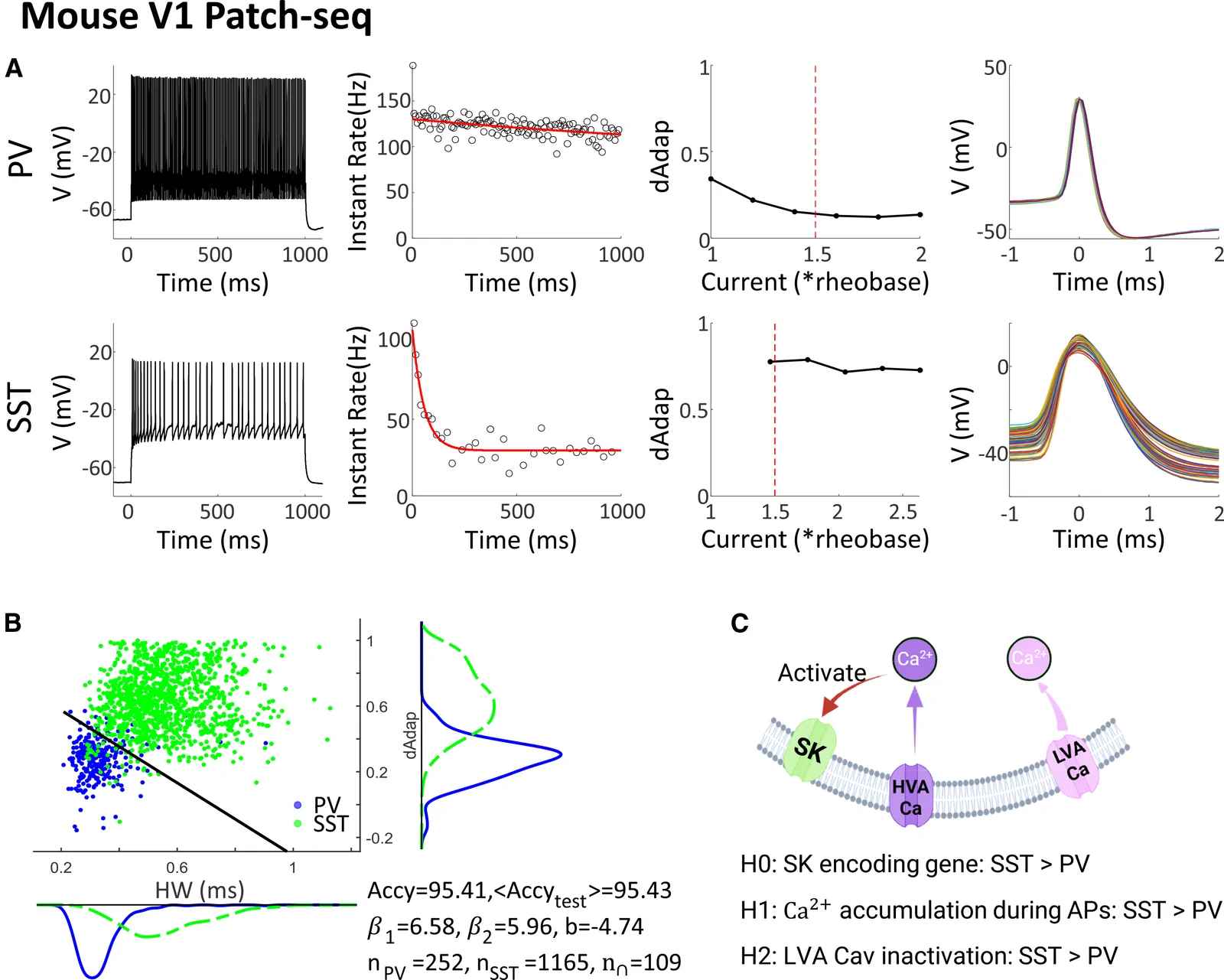
The degree of adaptation and the width of the action potential are highly correlated (A) Different electrophysiological features of PV and SST INs. From left to right: one recording over a 1 s-long square-pulse current injection. Instantaneous firing rate as one over the interspike intervals. The curve is fitted to an exponential decay function. dAdap over injection currents. The red dashed line indicates 1.5× the rheobase. AP shapes are overlaid, while each line represents an AP from the PV cells. (Top) Results from an example PV cell; bottom, an SST cell. (B) PV and SST cells can be classified based on HW and dAdap. The dashed line is the classification boundary trained by a support vector machine, which is indicated by *y* = −(*xβ*_1_ + *b*)/*β*_2_. See Table S1 for details. Accy, classification accuracy, tested accuracy < Accy_*test*_ > is calculated as the average performance when trained on a randomly split 80% training dataset and tested on the remaining 20% testing set. The sample sizes of PV cells, SST cells, and both *Pvalb*+/*Sst*+ cells (see the main text) are indicated by *n*_PV_, *n*_SST_, and *n*_∩_. (C) Three hypotheses that may explain the observed firing rate adaptation. The stronger adaptation in SST INs may result from H0: increased SK channel conductance directly mediating mAHP; H1: greater per-spike *Ca*^2+^ influx that activates SK channels; H2: enhanced inactivation of LVA Cav channels on a fast timescale; or a combination of these mechanisms.

We quantitatively measured the firing-rate adaptation by the degree of adaptation (dAdap). To do so, we first fit the curve of the instantaneous firing (IF) rate to an exponential decay function *f*(*t*) = *a* + *b* exp(−*ct*). After fitting, we compare the firing rate at the end of the 1*s* simulation to the initial firing rate dAdap = 1 − *f*(1)/*f*(0), which varies little at high injection current (above 1.5× rheobase, Figure 1A, 3rd column). In the following analysis, we use the closest sweep above 1.5× rheobase to measure the dAdap for each cell. We quantify the AP waveform by HW, the average time above half of the peak, and the firing threshold for each AP (see STAR Methods).

Next, we investigate how effectively HW and dAdap can distinguish PV and SST cells, compared to other classification methodologies. In,^25^ PV and SST cell types are defined through a multi-gene classification algorithm. Based on HW and dAdap along, a trained classifier achieves an accuracy of 95.3% (Figure 1B). Conventionally, classification is performed using marker genes, such as *Pvalb* and *Sst*, like through immunohistochemistry labeling. To compare the effectiveness of multi-gene classification and single marker gene classification, we analyzed the marker gene distribution in the V1 dataset (Figure S1A). Assuming a threshold at the 20th percentile of *Pvalb* and *Sst* counts per million (CPM) distribution to determine whether a cell is *Pvalb*+ or *Sst*+, respectively, we identified a significant population of *Pvalb*+/*Sst*+ cells, resulting in an 89.6% agreement with the multi-gene classification (Figure S1A). This suggests that a classifier based on HW and dAdap is better aligned with the multi-gene classification than one based on the conventional marker genes. In addition, we did not find a significant difference in classification accuracy when considering laminar differences (Figure S1B). Moreover, our trained classifier from V1 achieves a 86.4% accuracy on a PV/SST dataset collected from L2/3 of the somatosensory area in mice (Figure S1C).

Since firing rate adaptation and AP half-width differ significantly between PV and SST interneurons, we next asked whether these physiological differences are reflected at the transcriptomic level. In particular, we focused on uncovering the molecular mechanisms underlying firing-rate adaptation, which may arise through distinct pathways (Figure 1C). In pyramidal cells, adaptation is typically attributed to a progressive increase in medium AHP (mAHP) across action potentials, driven by accumulating intracellular calcium.^22^^,^^27^ Based on this, our default hypothesis (H0) is that SST cells express more SK channel encoding genes than PV cells. However, since SK channel activation depends on calcium influx per spike, an alternative hypothesis (H1) is that differences in calcium entry per AP may modulate SK activation. This calcium entry is shaped by the time window for the calcium flux and the flow rate, related to HW and HVA Cav conductance, respectively. Finally, we propose (H2) low-voltage-activated (LVA) calcium channels, which can inactivate rapidly, may also contribute directly to firing rate adaptation at a fast timescale.

### dAdap differences are better reflected in the calcium influx related genes

Since HW and dAdap differ significantly between PV and SST populations, we next investigated the underlying mechanisms and whether they are reflected in transcriptomic data. We began by performing quality control (Figures 2A and 2B), including only cells with high total raw counts and numbers of unique detected genes. Additionally, patch-seq data may include contamination from nearby microglia.^30^ Following the approach in the study by Tripathy et al.,^30^ we computed a contamination score for each cell and excluded those with high scores (Figure 2B; see STAR Methods). To ensure reliable dAdap measurements, we also excluded cells not driven to at least 1.5 times rheobase. Threshold variation within a reasonable range yielded qualitatively similar results (Figure S2). After all quality control steps, approximately 35% of cells remained. We further excluded genes with low expression (see STAR Methods). In subsequent analyses, genes within a family of interest may be omitted if they did not pass quality control.

**Figure 2:**
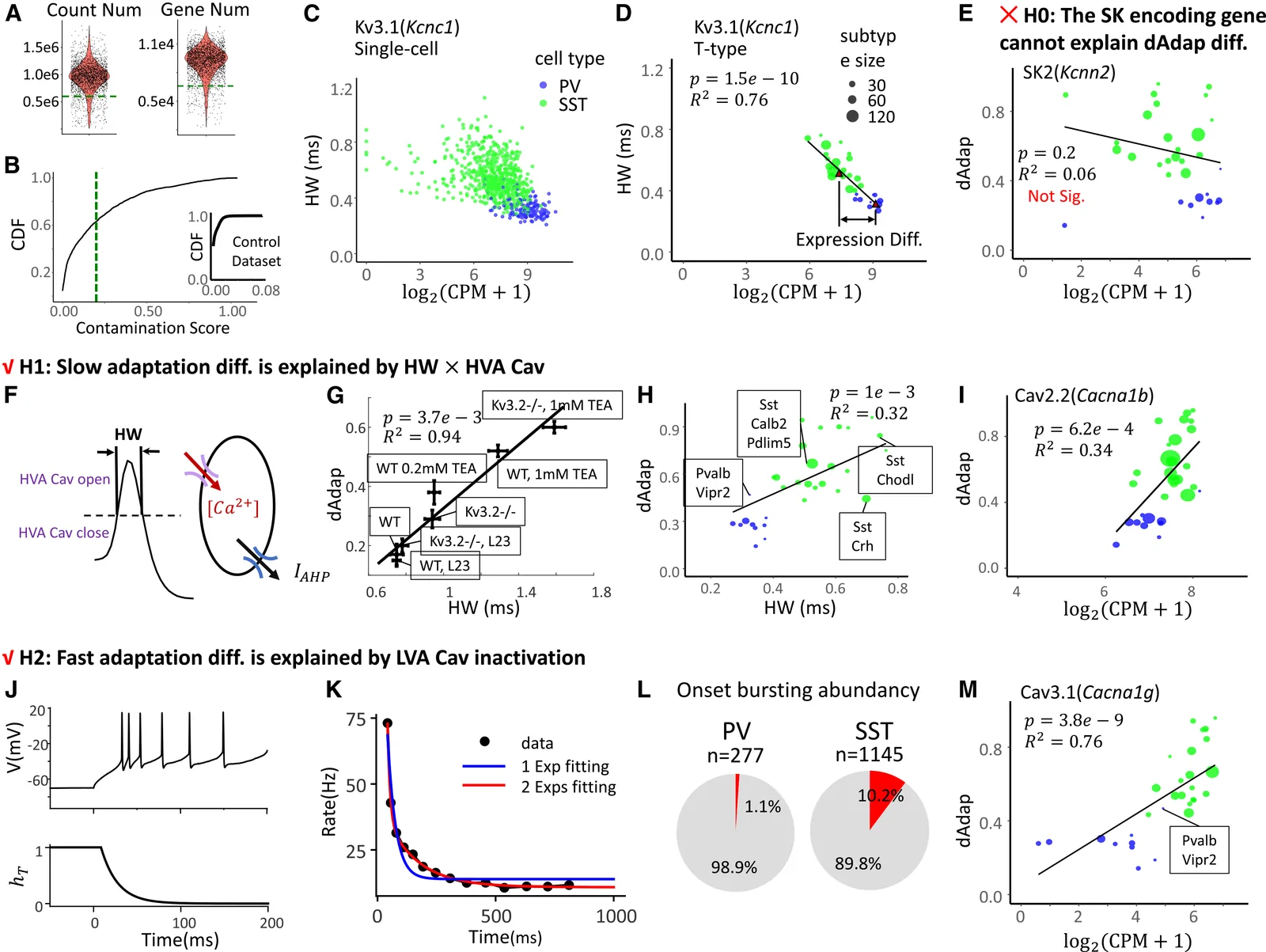
dAdap differences are reflected in the expression of calcium influx-related genes, but not SK channel-encoding genes (A) Quality control of patch-seq V1 scRNA-seq data. Dashed lines indicate the filtering thresholds, set just above the tail of the distribution: 6 × 10^5^ (left) and 6, 600 (right). (B) Cumulative distribution function (CDF) of the contamination score (CS; see STAR Methods). Cells with CS > 0.2 (dashed line) were excluded. Inset: CS distribution from a dissociation-based dataset,^28^ where all cells had CS < 0.08. (C) Scatterplot of spike half-width (HW) versus expression of *Kcnc1* (encoding Kv3.1) across individual V1 PV and SST cells. (D) Bubble plot showing T-type-averaged values from (C). The cell-type expression difference is shown by arrows. Dot size indicates sample size; the solid line denotes the weighted linear regression (WLR) fit. *p*: *p* value; *R*^2^: proportion of variance explained. (E) Bubble plot of dAdap versus expression of *Kcnn2*. The correlation is not significant, which does not agree with H0. (F–I) Results supporting H1. (F) Schematic of the HVA Cav channel mechanism underlying SRA. During each AP, HVA Cav channels open upon sufficient depolarization, with the activation window approximately reflected by HW. The resulting *Ca*^2+^ influx activates SK channels, generating an outward AHP current that contributes to SRA. (G) Increased HW is associated with stronger adaptation. Both HW and dAdap increase following Kv3.2 gene knockout or Kv3 channel inhibition using TEA. Error bars indicate SEM. Data reanalyzed from,^29^ collected from layer 5/6 mouse M1 fast-spiking interneurons of either sex at room temperature, unless otherwise noted. (H) Bubble plot of T-type-averaged HW and dAdap in V1. T-type annotation follows.^25^ (I) Bubble plot of dAdap versus expression of *Cacna1b*. (J–M) Results supporting H2. (J) Voltage depolarization from the current step rapidly inactivates LVA Cav channels, reducing total inward current. Top, zoomed-in voltage trace from an example SST cell. Bottom, inactivation of the gating variable *h*_*T*_ of LVA Cav channels. (K) Instantaneous firing (IF) rate of an SST cell exhibiting onset-bursting (OB). The IF curve is fitted with either one (blue) or two (red) exponentially decaying components. The two-component fit provides a better match in this case. (L) OB is more prevalent in SST cells than in PV cells. (M) Bubble plot of dAdap versus expression of *Cacna1g*.

Previous work has shown that HW is controlled by voltage-gated K^+^ (Kv3) and Na^+^ (Nav) channels in INs.^31^^,^^32^ Specifically, higher Kv3 conductance leads to faster repolarization after each spike, resulting in a narrower AP waveform and smaller HW. At the single-cell level, greater expression of *Kcnc1* (encoding Kv3.1) is correlated with smaller HW (Figure 2C), though with substantial variability. Some cells may show zero *Kcnc1* expression due to dropout, where no transcript is detected despite low expression. To enhance signal and mitigate dropout effects, we aggregated cells at the transcriptomically defined type (T-type) level. These T-types, defined via unsupervised clustering across all genes, exhibit consistent electrophysiological and morphological features.^25^ Weighted linear regression (WLR) at the T-type level reveals a strong correlation between *Kcnc1* expression and HW (Figure 2D, *p* = 1.5 × 10^−10^). These findings are consistent with prior studies^31^^,^^32^ and support the validity of our approach in linking electrophysiological features with transcriptomic data.

Having validated our methodology with HW-related genes, we next examined genes related to dAdap. The default mechanism (H0) to explain the observed dAdap difference is that SST cells express more SK channel encoding genes than PV cells. However, we found no significant correlation between dAdap and corresponding encoding genes (Kcnn2, Figure 2E; Kcnn1 and 3, see in further text), which does not agree with the default hypothesis.

Next, we ask whether the (H1) calcium inlux related mechanisms are reflected in the transcriptomic data (Figure 2F). That is, even if SK conductance is similar between PV and SST cells, its activation may differ depending on the amount of Ca^2+^ influx during each AP (Figure 2F). This influx is determined by both the conductance of Cav channels and the duration of their activation. The latter can be approximated by HW, while the former should be reflected in the expression of the corresponding Cav channel genes. To test H1, we reanalyze data from the study by Lau et al. and Erisir et al.,^29^^,^^33^ which show that manipulating HW, via Kv3.2 knockout or tetraethylammonium (TEA) treatment (an inhibitor of the Kv3 family), modulates SRA (Figure 2G). Consistent with this, HW and dAdap are significantly correlated across SST and PV T-types (Figure 2H), and *Kcnc1* (encodes Kv3.1) is also significantly correlated with dAdap (Figure 2I).

In addition, (H2) inactivation of LVA Cav channels may also contribute to dAdap on a faster timescale (Figure 2J). Indeed, IF rate curves from some SST cells are better fit by two exponential components rather than a single one, while the latter is the standard for quantifying dAdap (Figure 2K). This fast timescale component can be captured by the initial slope of the IF curve (Figure S3B). We further classified a cell as having an onset bursting (OB) feature if the fast component is statistically significant and its rectified amplitude exceeds 10 Hz (see STAR Methods). Approximately 10% of SST cells exhibit OB, whereas only a few PV cells (1.1%) do (Figure 2L). These OB-positive SST cells are enriched in specific T-types (Figure S3E). Consistent with this, SST T-types express more *Cacna1g* (encoding LVA T-type Ca channel) than PV T-types (Figure 2M; Figure S2F), aligning with previous findings.^34^

However, our method is limited to qualitatively identifying OB based on the current data, rather than providing robust quantitative estimates. Typically, only about 3 APs contribute meaningfully to fitting the fast component. Consequently, while the presence or absence of an OB feature can be assessed statistically (see STAR Methods), the amplitude of the fast component exhibits high variability (Figures S3C and S3D).

Among the T-types, *Pvalb Vipr2*, which corresponds to chandelier cells, shows elevated dAdap (Figure 2H), consistent with findings from.^35^ This T-type also displays strong *Cacna1g* expression (Figure 2M) and includes a cell exhibiting the OB feature (Table S1).

In addition to the MGE-derived PV and SST interneurons, we further investigated whether our findings extend to CGE-derived subtypes, including VIP, SNCG, and LAMP5 INs (Figure S4), all collected from the same patch-seq dataset.^25^ These CGE-derived INs exhibit more complex firing patterns beyond simple spike-rate adaptation (SRA), including acceleration, strong OB, and irregular firing,^36^ which can confound the dAdap measurement. To address this, we restricted our analysis to cells that passed the quality control for dAdap and excluded cells exhibiting OB features across both MGE- and CGE-derived INs (see STAR Methods). While this approach helps minimize measurement artifacts, it reduces the available sample size and may introduce selection bias.

Given this, we next incorporated VIP (*n* = 38) and SNCG (*n* = 11) interneurons into the analysis (Figures S4A–S4L). When analyzing all four subtypes together—PV, SST, VIP, and SNCG—we found that genes related to upstream calcium influx and LVA Ca channels remained significantly correlated with dAdap, consistent with the conclusions drawn from PV and SST cells alone. Although Kcnn2 expression was not broadly correlated with dAdap across all interneurons (Figure S4C), CGE-derived VIP and SNCG cells showed significantly higher expression of *Kcnn2* compared to MGE-derived PV and SST cells (Figure S4I). This is in line with the observation that CGE-derived INs exhibit stronger dAdap than MGE-derived INs (Figure S4G), suggesting that the differences in adaptation across all IN subtypes may reflect a composite effect involving all three hypothesized mechanisms.

However, our proposed mechanisms do not account for the observations in LAMP5 interneurons (*n* = 81). LAMP5 cells, which also express genetic markers such as *Ndnf* and *Id2*,^28^^,^^37^ are primarily considered as late-spiking neurogliaform INs. These cells exhibit broader action potentials but reduced dAdap, comparable to that of SST, VIP, and SNCG interneurons (Figure S4G). Despite this, expression levels of *Kcnn2*, *Kcnc1*, HVA, and LVA Cav channel encoding genes of LAMP5 cells are similar to those of VIP and SNCG cells (Figures S4G–S4L), a profile inconsistent with any of our current hypotheses.

Notably, LAMP5 cells exhibit late spiking and accelerating firing near rheobase,^36^^,^^37^ suggesting the presence of a slowly activating inward or inactivating outward current during the depolarizing step. We speculate that this current may underlie the unique electrophysiological profile of LAMP5 cells compared to other MGE- and CGE-derived interneurons. However, elucidating the exact ionic mechanisms is beyond the scope of the current study.

### dAdap differences are explained by a combination of gene families

We next investigate gene families relevant to predicting HW and dAdap, along with their relative importance. We begin by ranking genes based on their correlation significance with HW and dAdap, while showing their expression difference between SST and PV cells (for one example, see Figure 2D). For HW (Figure 3A), the Nav and Kv3 families showed the strongest correlation, consistent with previous findings.^31^^,^^32^ The corresponding differential expression (DE) analysis is presented in Figure S5A. Additionally, we find that the expression levels of several genes are highly correlated across IN T-types and can be approximately separated into two anti-correlated groups (Figure S5B). This suggests that these genes may vary in a coordinated manner as part of broader functional gene-expression modules.^38^ Moreover, correlation significance is linearly related to the variance explained in the data (Figure S5C).

**Figure 3:**
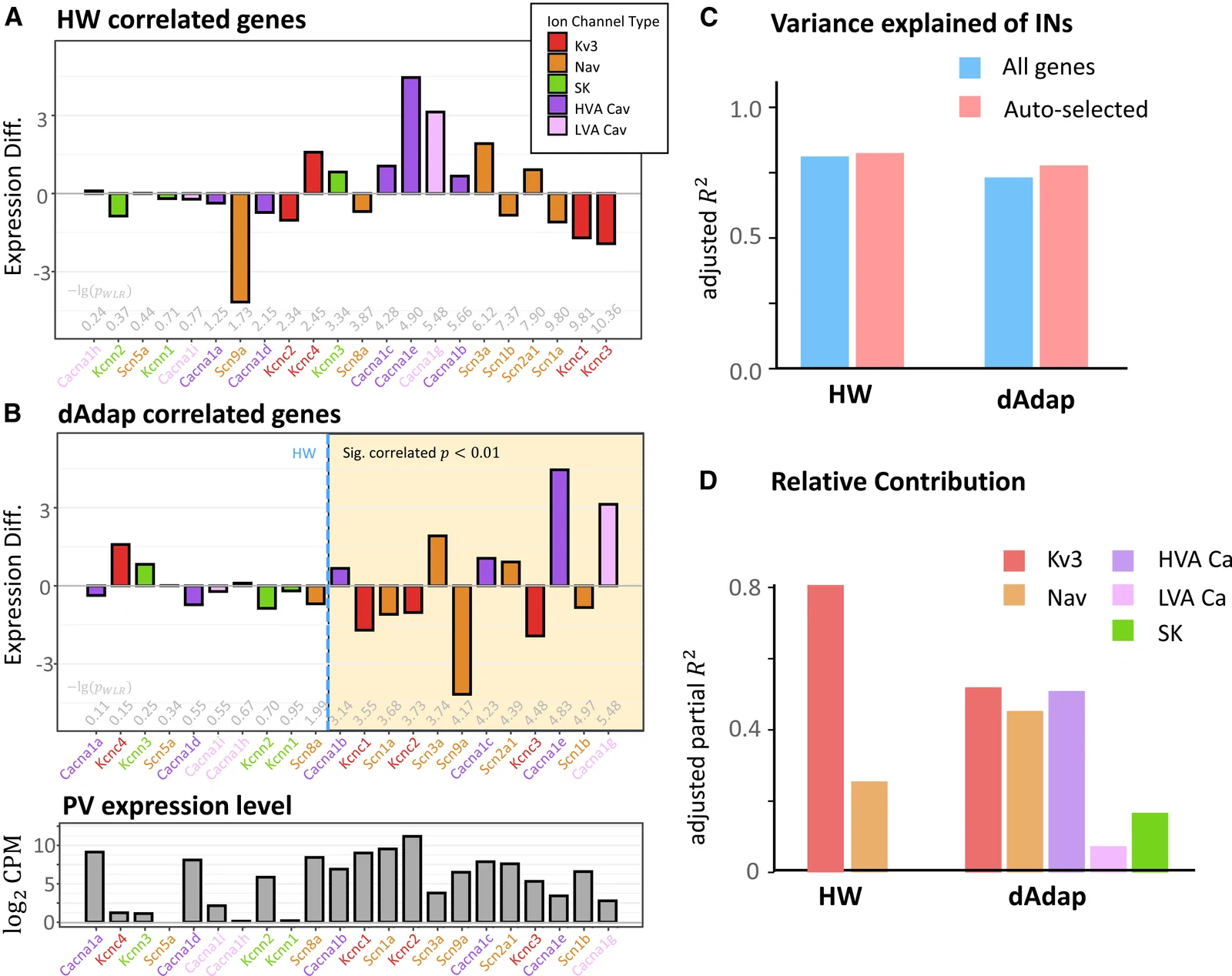
dAdap differences are explained by a combination of gene families (A) Ranked genes correlated with HW. Genes to the right show stronger correlations with HW, quantified by *p*_WLR_. Significance values (−log_10_(*p*_WLR_)) are annotated in gray. Bar heights represent the expression difference, measured by log_2_ fold change in SST cells relative to PV cells. Color indicates ion channel family. The black dashed line marks *p*_WLR_ < 0.01. (B) Top, ranked genes correlated with dAdap. The blue dashed line indicates the correlation significance for HW. Bottom, mean gene expression in PV cells. (C and D) Regression models for PV, SST, VIP, and SNCG INs. (C) Bar plot of adjusted *R*^2^ from multivariate regression models predicting HW or dAdap from candidate genes. Blue bars: models including all related genes and a class-dependent variable (HW: 80.9%, dAdap: 72.8%; see STAR Methods). Pink bars: models based on auto-selected genes (HW: 82.1%, dAdap: 77.4%). (D) Relative contribution of each ion channel family, quantified by adjusted partial *R*^2^ (see STAR Methods). See Figure S7 and Table S2 for further details.

Previous work has suggested that SRA is primarily mediated by SK channels.^22^ However, none of the SK channel-encoding genes show significant correlation with dAdap differences (Figure 3B, green). In contrast, HW itself (Figure 3B, blue dashed line), HW-related genes (Nav and Kv3 families; orange), and several Cav channel-encoding genes (Figure 3B, purple and pink) are significantly correlated with dAdap. Genes significantly correlated with dAdap tend to be highly expressed in both PV and SST interneurons (Figure 3B, bottom). However, some genes are highly expressed in PV cells yet do not correlate with dAdap differences. For example, *Kcnn2* is highly expressed in PV cells (Figure 3B, bottom), again suggesting the presence of SK channels, but they may not be sufficiently activated by Ca^2+^ influx through HVA Cav channels. Similarly, *Cacna1a* is strongly expressed in both PV and SST cells (Figure 3C). Still, it does not correlate with dAdap, highlighting that a gene may be functionally essential for the mechanism yet not contribute to dAdap variability across cell types. These trends remain consistent when analyzing gene-dAdap correlations at the single-cell level instead of the T-type level (Figures S6A and S6B), when excluding cells with OB features (Figures S6C and S6D), or when removing T-types where over one-third of cells exhibit OB features (Figures S6E and S6F). Interestingly, the LVA Cav gene *Cacna1g* still shows significant correlation with dAdap even after excluding OB cells and OB-enriched T-types (Figure S6). One possibility is that a small LVA Ca^2+^ current contributes to dAdap but is too weak to generate a significant OB signature detectable by our method.

To evaluate the collective contribution of gene families, we construct multi-gene WLR models including all significantly contributing families (Nav and Kv3 for HW; Nav, Kv3, HVA Cav, LVA Cav, and SK for dAdap). Since the subunits encoded by SK-family genes can form a heterotetramer,^39^^,^^40^ along with the fact that *Kcnn2* expression is anti-correlated with that of *Kcnn3* (Figure S4B), we use the summed SK expression values for each T-type as a single independent variable. We perform the regression in the case that includes PV, SST, VIP, and SNCG INs (Figures 3C and 3D, Figures S7E and S7F), and the case only of PV and SST INs (Figures S7A–S7D).

Together, these genes explain over 90% of the variance (Table S2). However, due to strong inter-gene correlations (Figure S5B), including all the genes has the risk of overfitting, as the number of predictors is high. To address this, we use adjusted *R*^2^, accounting for model complexity, and implement an auto-selection procedure to retain only the most informative genes (Figure 3C; see STAR Methods). This reduced model improves the adjusted *R*^2^ relative to the full model (Figure 3C).

When we analyze the case that only includes PV and SST, we also include a continuous cell-type variable defined by the log_2_ fold change between *Pvalb* and *Sst* expression to test the potential class-dependent effect (Figures S7A–S7D; see STAR Methods). Notably, the continuous cell-type variable is excluded during auto-selection, suggesting minimal cell-type-specific effects in the PV and SST dataset.

Based on the auto-selected model, we quantify the relative importance of each gene family using adjusted partial *R*^2^ (Figure 3D, Figure S5B; see STAR Methods). This analysis confirms that all included gene families contribute meaningfully to explaining the observed differences in HW and dAdap. Notably, the LVA Ca^2+^ family shows the lowest explanatory power, consistent with the observation that only about 10% of SST cells exhibit the OB feature.

We further analyze the contribution of individual genes using adjusted partial *R*^2^ (Figures S5C–S5F). This gene-level analysis yields results that are qualitatively consistent with the gene-family-level analysis, indicating that the main conclusions are not driven by the use of aggregated gene-family expression alone. Among LVA Ca^2+^ channel-encoding genes, the only gene retained in the auto-selected model is *Cacna1h*, rather than *Cacna1g*, and its adjusted partial *R*^2^ is limited, at approximately 0.1. This suggests that individual LVA Ca^2+^ channel genes provide only a modest contribution to the statistical prediction of dAdap. However, given the correlation structure among individual genes (Figure S5B), gene-expression information is partly redundant across model features, which should be taken into consideration when interpreting the importance of individual genes.

For SK channel-encoding genes, because SK channels can form heterotetramers, we use the summed expression of SK-family genes as a single independent variable rather than treating each SK gene independently. Another important point is that although the SK gene family remains in the auto-selected model, its regression coefficient is negative in both the four-IN subtype analysis and the PV/SST-only model (Figures S7D and S7F), which is inconsistent with known SK channel biology. Therefore, this finding cannot be considered support for the default hypothesis (H0). Instead, it suggests that SK gene-expression differences may contribute statistically to model performance but do not provide a biologically interpretable explanation for the stronger dAdap observed in SST cells compared with PV cells.

Additionally, *Cacna1e*, which encodes the R-type Cav channel (Cav2.3), is significantly correlated with dAdap (Figure 3B; Figure S5F), and shows a comparable contribution to other HVA Cav encoding genes (*Cacna1a,b* in Figure S7C, and *Cacna1a-d* in Figure S7E). This broader association across multiple HVA Cav channel-encoding genes is consistent with the established role of HVA Cav channels in activity-dependent Ca^2+^ influx and Ca^2+^-dependent spike-frequency adaptation mechanisms. In particular, prior work has shown that several non-R-type HVA Cav channels, including N-type and L-type channels encoded by *Cacna1b* and *Cacna1c*, can contribute to SK-channel-dependent mAHP.^41^ However, the same study^41^ showed that R-type Cav channels may not activate SK channels, suggesting that the observed correlation between *Cacna1e* and dAdap specifically may reflect an indirect or non-causal correlation.

In the following analysis, we focus on comparing PV and SST INs for three reasons. First, the auto-selected models yield similar results when comparing all four subtypes versus PV and SST only. Second, the VIP and SNCG datasets are limited in size and may introduce sampling bias. Third, MGE-derived PV and SST interneurons together comprise 70% of all cortical INs and represent the primary inhibitory input to pyramidal neurons.^19^

### Other potential genes

Beyond genes directly linked to our hypothesized mechanisms, other genes may also modulate dAdap. To identify such candidates, we examine differentially expressed genes (DEGs) between PV and SST INs and assess their correlation with dAdap or HW using WLR (Figure 4A; see STAR Methods). In our experience, DEG analysis highlights genes with large fold changes between PV and SST cell types, whereas WLR focuses on whether relative gene expression trends explain observed electrophysiological differences. In total, 322 DEGs are significantly correlated with dAdap; among them, 269 also correlate with HW, while 53 do not.

**Figure 4:**
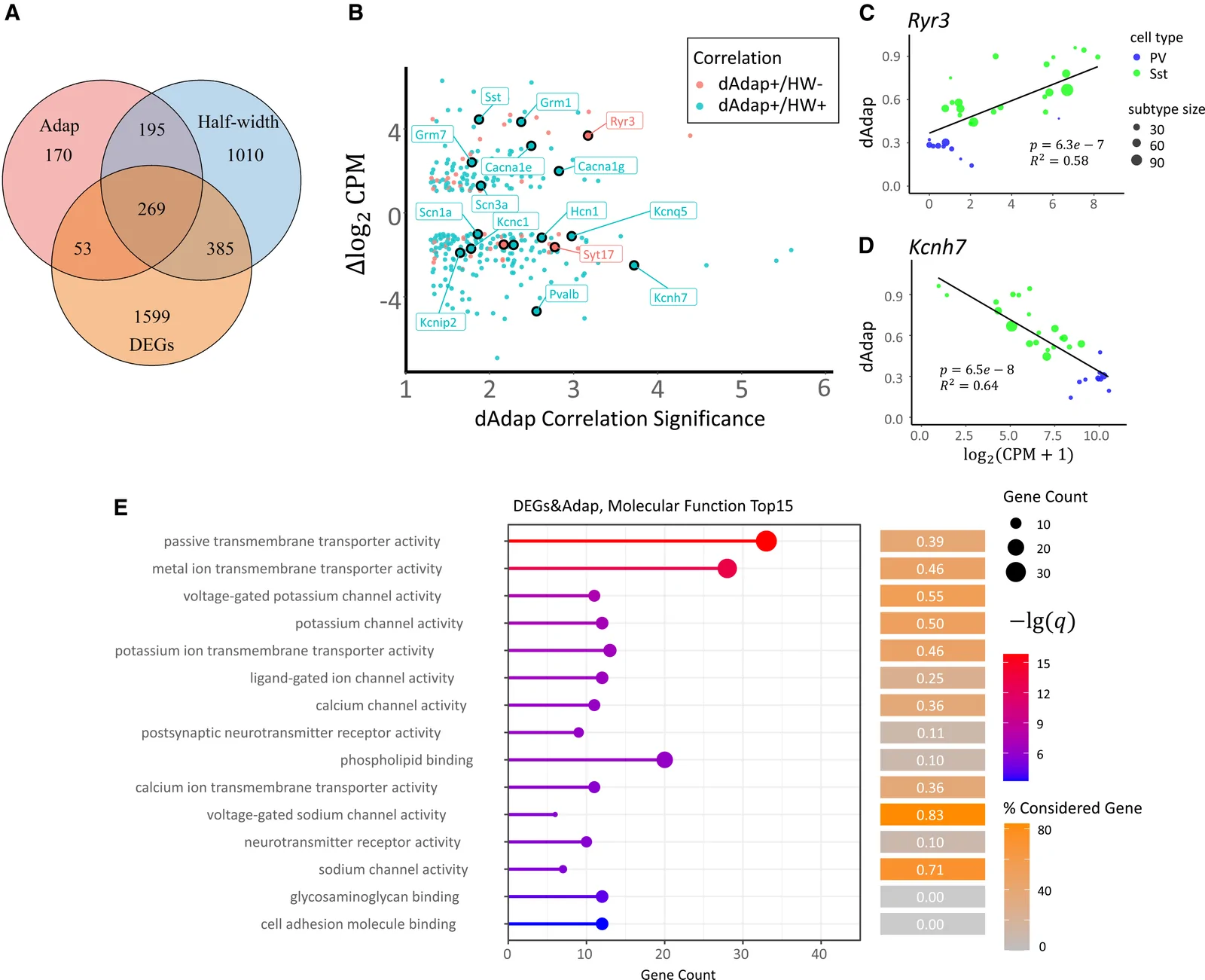
List of potential contributing genes generated by an unsupervised filtering algorithm (A) Venn plot of genes highly correlated with dAdap (pink), genes highly correlated with HW (blue), and differentially expressed genes (DEGs; orange). (B) Scatterplot of filtered genes. The *x* axis indicates the significance of the correlation with dAdap, measured by −log_10_(*q*_WLR_). The *y* axis indicates the fold change relative to PV cells. Genes highly correlated with both dAdap and HW are labeled in blue, whereas those highly correlated only with dAdap are labeled in pink. Here, we focus on genes with *q*_WLR_ < 0.05 and |Δ log_2_(CPM)| > 1. (C) Correlation between dAdap and *Ryr3*. (D) Correlation between dAdap and *Kcnh7*. (E) Gene Ontology enrichment analysis of molecular functions. The top 15 terms enriched among genes that are differentially expressed and significantly correlated with dAdap are shown. Colored cubes on the right indicate the proportion of genes discussed within each functional group. See Table S3 for details.

We further examine the fold change in SST versus PV cells and the false discovery rate (*q*_WLR_) for these 322 genes (Figure 4B). Consistent with our hypothesis, the 269 overlapping genes (blue points in Figure 4B) may influence dAdap by modulating the Ca^2+^ influx window via effects on HW. In contrast, the remaining 53 genes likely affect dAdap by altering Ca^2+^ flow rate or the sensitivity of SK channels to Ca^2+^. However, we cannot fully exclude the possibility that some of these genes are simply PV or SST marker genes, such as *Pvalb* and *Sst*.

Among the resulting gene list (Figure 4B), we identify our hypothesized genes *Kcnc1*, *Kcnc3*, *Scn1a*, and *Cacna1g*, supporting the validity of our unsupervised filtering approach. Additionally, *Ryr3*, which encodes the type 3 ryanodine receptor, is significantly more expressed in SST cells (Figure 4C) and is positively correlated with dAdap, but not with HW. Higher *Ryr3* expression has been shown to enhance Ca^2+^ release from intracellular stores,^42^ potentially increasing SK channel activation.^22^ Other candidate genes include *Grm1*, encoding a group I metabotropic glutamate receptor known to facilitate intracellular Ca^2+^ release, and *Syt17*, which may enable Ca^2+^ binding activity (Figure S8A).

We also identify several K^+^ channel-encoding genes: *Kcnh7*, *Kcnq5*, and *Kcnip2*, which are significantly more expressed in PV cells and negatively correlated with both HW and dAdap (Figure 4D; Figures S8B and S8C). These genes may modulate interneuron excitability.^43^ Among them, *Kcnq5* encodes the M-type K^+^ channel, which has been implicated in SRA in CA1 pyramidal neurons.^24^ However, if the M-type channel were a primary driver of dAdap, higher *Kcnq5* expression would be expected to associate with greater adaptation. This is contradicted by our data, which show that PV neurons exhibit higher *Kcnq5* expression yet smaller dAdap (Figure 4D).

BK channels may also contribute to the regulation of HW and dAdap (Figures S8D and S8E), consistent with previous findings.^44^^,^^45^ We also observe that the H-current-encoding gene *Hcn1* is significantly differentially expressed between PV and SST cells, although the fold change is close to one. Given that the H-current is activated by hyperpolarization, it is unlikely to influence HW or dAdap under conditions where neurons remain predominantly depolarized.

Finally, the SK channel function is modulated by accessory subunits, including calmodulin, protein kinase CK2, and protein phosphatase 2 A.^22^ However, the corresponding genes (*Calm1–3*, *Csnk2a1*, *Csnk2a2*, *Csnk2b*, *Ppp2r1a*, *Ppr2r1b*, *Ppp2ca*, and *Ppp2cb*) are not DEGs (see Table S3). Thus, these factors are unlikely to explain dAdap differences between cell types.

We perform Gene Ontology (GO) enrichment analysis^46^^,^^47^ to identify shared functional roles among DEGs, providing biological insight into underlying mechanisms. The GO analysis on our interest 322 DEGs (Figure 4E) reveals enrichment in ion transmembrane transporter activity. Particularly K^+^, Ca^2+^, and Na^+^ channels, which are consistent with our hypothesis. A large proportion of genes identified in the GO analysis have already been discussed (Figure 4E, colored cubes). To the best of our knowledge, the remaining genes among the top 15 GO terms do not show clear functional relevance to SRA. The complete gene list from the GO analysis is provided in Table S4.

### Validation in an M1 patch-seq dataset

We next ask whether our hypotheses generalize to other brain regions. To test this, we analyze a patch-seq dataset from the mouse primary motor cortex (M1), in which electrophysiological features were recorded at room temperature.^26^ A significant correlation between HW and dAdap is observed (Figure 5A).

**Figure 5:**
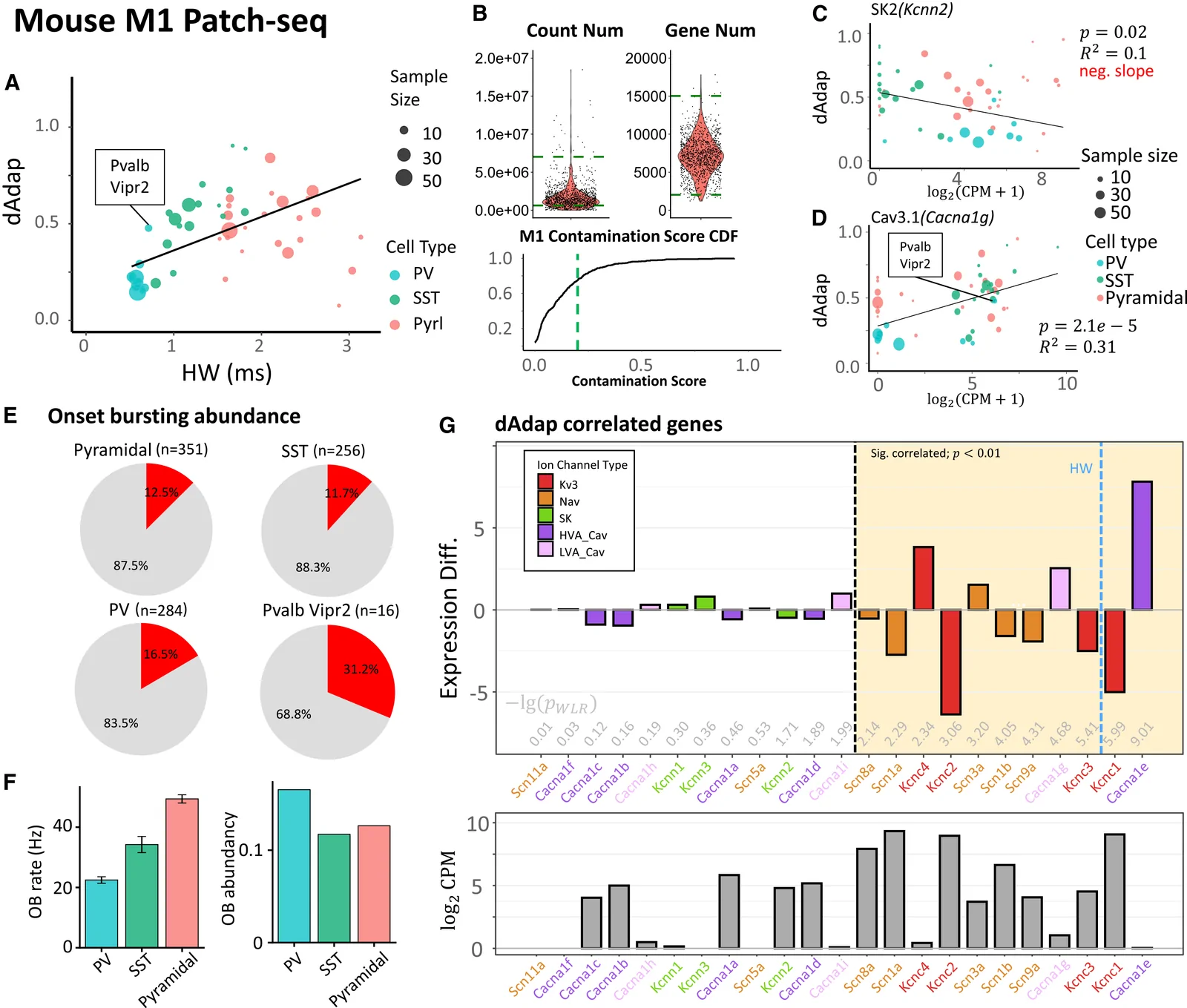
Validation in a mouse M1 dataset (A) Correlation between HW and dAdap at the T-type level in the mouse M1 dataset. The dashed line indicates the fit for PV and SST INs only. *Pvalb Vipr2* corresponds to chandelier cells. (B) Quality control based on total gene count, gene number, and contamination. (C) Bubble plot of T-type-averaged dAdap and expression of the SK2-encoding gene *Kcnn2* in M1 pyramidal, PV, and SST cells. (D) Bubble plot of dAdap and expression of the Cav3.1-encoding gene *Cacna1g*. (E) Pie charts showing OB abundance in M1. (F) Bar plots of OB rate (left) and OB abundance (right). OB rate measurements are subject to large variance. See Figure S3. (G) Top, ranked genes correlated with dAdap. Bar heights indicate expression differences, measured as the log_2_ fold change in pyramidal cells relative to PV cells. Significance, measured as −log_10_(*p*_WLR_), is annotated in gray. The black dashed line indicates *p*_WLR_ < 0.01. The blue dashed line indicates the significance of the correlation with HW. Bottom, mean gene expression in PV cells. See Table S6 for details.

After applying similar quality control procedures (Figure 5B) and performing DE analysis (Figure S9A), we find results qualitatively consistent with the V1 dataset. Specifically, dAdap is not correlated with the SK channel gene *Kcnn2* (Figure 5C), but is correlated with HW-related genes and Cav genes, including *Cacna1g* (Figure 5D). A subset of pyramidal, SST, and PV cells exhibited OB features (Figure 5E). Interestingly, a larger fraction of PV cells displayed OB features compared to the V1 dataset, although the amplitude of the fast component (measured by OB rate) is relatively small (Figure 5F). One potential reason is that neurons in the M1 dataset were more commonly recorded at higher depolarizing currents (e.g., four times the rheobase), making the small-amplitude fast component easier to detect in sweeps with more action potentials. However, differences in recording temperature and brain region may also contribute. The precise mechanism requires further investigation. Some PV T-types (referred to as RNA-types in the study by Scala et al.^26^) were enriched for OB cells. Among them, the *Pvalb Vipr2* T-type, putative chandelier cells, is notably enriched in OB features, consistent with its high *Cacna1g* expression (Figure 5D).

We further analyze which genes are significantly correlated with HW or dAdap (Figure S9B; Figure 5G). HW correlates with known genes such as *Kcnc1*, *Kcnc2*, and *Scn1a* (Figures S9B–S9D), while dAdap correlates with the same HW-related genes and the Cav gene *Cacna1g*, but not with SK channel genes *Kcnn1*–*Kcnn3* (Figure 5G). In addition, *Cacna1e* (Cav2.3, R-type) is significantly correlated with both HW and dAdap, consistent with the previous observation in V1.

However, when analyzing HW and dAdap differences within interneurons and pyramidal cells separately, distinct mechanisms may emerge. This phenomenon is known as the class-driven effect.^7^ For instance, across the combined population of excitatory (E) and inhibitory (I) cells, *Kcnc1* and *Scn1a* show a negative correlation with HW (Figures S9C and S9D), consistent with the idea that increased K^+^ or Na^+^ current accelerates repolarization or depolarization, leading to narrower AP waveforms. However, when restricting the analysis to either E or I cells alone, this negative correlation persists only within the I population (Figures S9E and S9F), highlighting potential class-specific regulatory mechanisms.

We quantify the class-driven effect using the significance of an interaction term in a statistical model, following^7^ (see STAR Methods). This effect is significant for *Kcnc1* in explaining HW differences (Figure S9E; *p*_class_ = 1.5 × 10^−8^), and is also observed for *Scn1a* (Figure S9F). The full list of genes with class-driven effects is provided in Table S5. Similar effects are observed for Cav and SK channel-encoding genes (Figures S9G and S9H).

We further investigate the mechanisms underlying HW differences in pyramidal cells. None of the Kv3 genes significantly explains the observed HW variability within this population (Figure S10A). Alternatively, BK channels have been proposed to regulate HW in pyramidal cells.^44^^,^^45^ However, our data do not reveal a significant negative correlation between BK channel-encoding genes and HW (Figures S10B and S10C). Given the low expression levels and pronounced dropout in these genes (Figures S10A and S10C), we suspect that the true signal may be obscured by noise, limiting detectability.

### Regional transcriptomic and electrophysiological differences across macaque monkey brain regions

To test whether our hypothesis generalizes across cell types, brain regions, and species, we analyze one electrophysiological and one transcriptomic dataset from macaque monkeys,^3^ covering the same three brain areas: V1, lateral intraparietal area (LIP), and prefrontal cortex (PFC). HW and dAdap are measured as before (Figures S11A and S11B), with a few cells exhibiting OB features (Figure S11C).

We first observe a significant correlation between HW and dAdap across individual cells from all three brain areas (Figure 6A). Within each region, both HW and dAdap are consistently lower in interneurons compared to excitatory cells. Across regions, HW increases in pyramidal cells from V1 to PFC, and is significantly higher in PFC than in V1 for interneurons (Figure 6B). Similarly, dAdap is higher in PFC than in V1 for both pyramidal cells and interneurons, though the difference is not statistically significant (Figure 6B).

**Figure 6:**
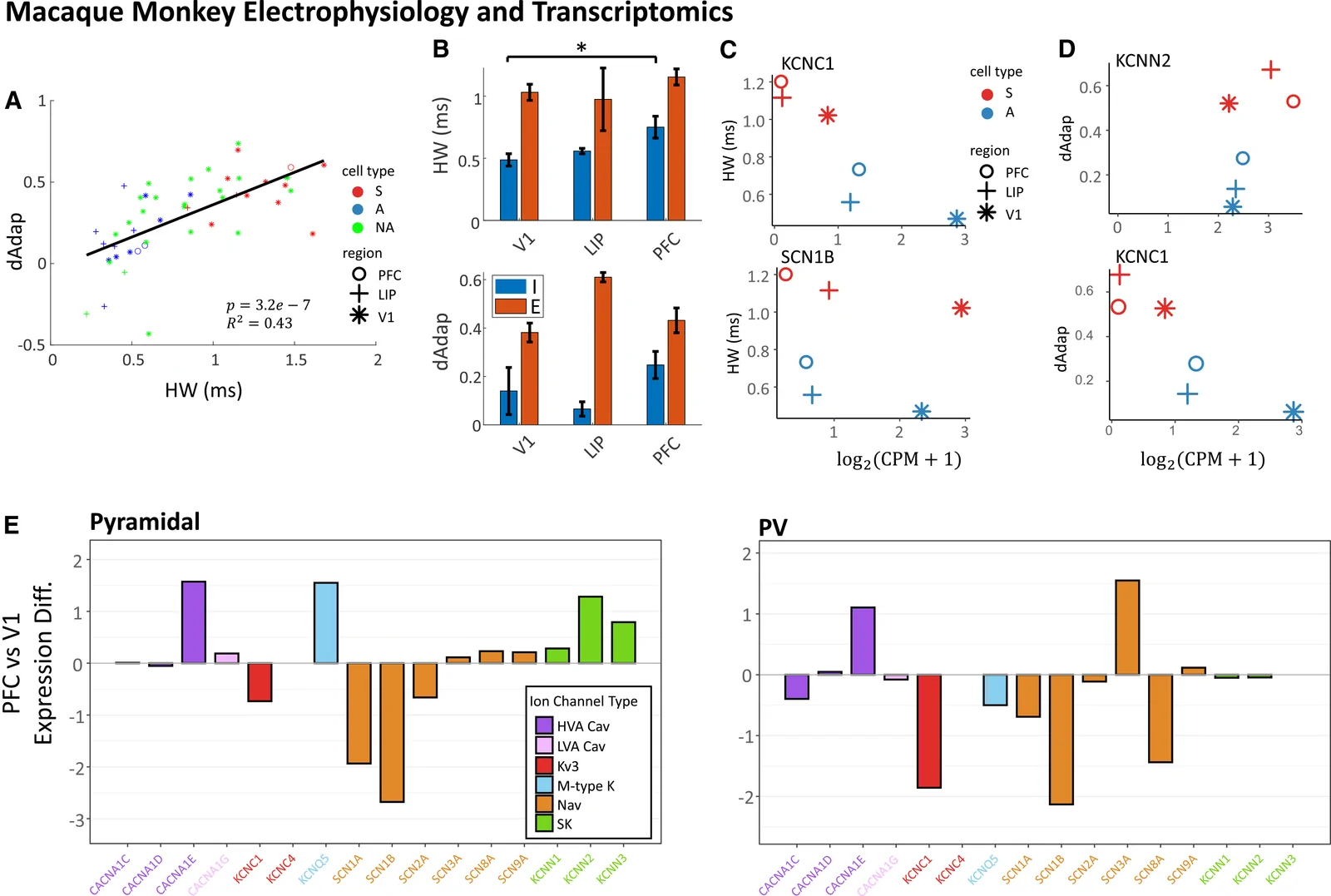
Cell-type and regional differences in macaque monkey datasets Transcriptomic data are from the study by Chen et al.^3^ (A) Correlation between HW and dAdap at the single-cell level in the multi-regional macaque dataset. Cell types are determined by cell morphology. A, aspiny, putative INs; S, spiny, putative excitatory pyramidal cells; NA, not applicable. Different symbols indicate the regions in which the recorded cells are located. *N* = 50. (B) Regional differences in HW (top) and dAdap (bottom) in the macaque dataset. ∗: *p* < 0.05. PFC INs have significantly wider APs and larger dAdap. *p*_HW_ = 0.02. Error bars indicate standard error. For HW, $N_{E}^{\text{V}1\text{,}\;\text{LIP,}\;\text{PFC}}=\left\{11,3,17\right\}$ and $N_{I}^{\text{V}1\text{,}\;\text{LIP,}\;\text{PFC}}=\left\{11,2,13\right\}$. For dAdap, $N_{E}^{\text{V}1\text{,}\;\text{LIP,}\;\text{PFC}}=\left\{2,2,10\right\}$ and $N_{I}^{\text{V}1\text{,}\;\text{LIP,}\;\text{PFC}}=\left\{6,3,12\right\}$. (C) Relationship between HW and expression of *KCNC1* and *SCN1B* across cell types and regions. A, aspiny, putative INs; S, spiny, putative excitatory pyramidal cells. Different symbols indicate the regions in which the recorded cells are located. (D) Same as (C), but for the relationship between dAdap and expression of *KCNN2* and *CACNA1E*. (E) Expression differences, measured by fold changes in gene expression relative to V1, in pyramidal cells (top) and PV cells (bottom). Ion channel families are color-labeled. $N_{\text{Pyr}}^{\text{V}1\text{,}\;\text{PFC}}=\left\{2.1\times 10^{3},1.3\times 10^{5}\right\}$ and $N_{\text{PV}}^{\text{V}1\text{,}\;\text{PFC}}=\left\{1.3\times 10^{3},1.1\times 10^{4}\right\}$. See Table S7 for the DEG analysis results.

We next asked whether the observed electrophysiological differences could be explained by transcriptomic data from the study by Chen et al.,^3^ which includes whole-brain macaque samples sequenced using unique molecular identifiers (UMIs) and clustered into cell types. For each subtype in each brain region, we calculated a trimmed mean expression. Similar gene expression patterns are observed across macaque PFC, LIP, and V1, consistent with the trends seen in mouse V1. For instance, HW differences are broadly reflected in the expression of *KCNC1* and *SCN1B* (Figure 6C). As in mice, dAdap differences are not explained by *KCNN2* but are instead associated with the HW-related gene *KCNC1* (Figure 6D). These findings suggest that the molecular mechanisms underlying HW and dAdap differences may be conserved between mice and macaques. The full DEG analysis is provided in Table S7.

When comparing across regions (Figure 6E), we find that in pyramidal cells, *KCNC1*, *SCN1A*, and *SCN1B* all show significant down-regulation in PFC compared to other areas, consistent with the wider HW observed electrophysiologically. Notably, *SCN1A* and *SCN1B* exhibit much larger fold changes than *KCNC1*, suggesting that regional HW differences in pyramidal cells may primarily arise from changes in Na^+^ channels rather than K^+^ channels. Interestingly, both M-type and SK channel-encoding genes show an increasing trend in PFC. Combined with the broader HW, our hypothesis predicts stronger SRA in this region, which aligns with the trend in Figure 6B. We also note increased expression of the M-type channel gene *KCNQ5* in PFC, suggesting a potential role in regional dAdap differences.

INs exhibit a significant increase in HW in PFC compared to V1. This difference could arise from either changes in cell-type composition (e.g., fewer PV cells and more SST cells) or intrinsic property changes across regions. The compositional explanation appears less likely, as PV cells consistently comprise 40%–50% of the combined PV and SST population across regions^3^ (though see in the study by Torres-Gomez et al.^21^ for contrasting findings). Nonetheless, single-cell properties may vary unevenly across types. In our analysis, *KCNC1*, *SCN1A*, and *SCN1B* (but see *SCN3A*) all show significant down-regulation in PV INs in PFC (Figure 6E, right), consistent with the broader HW observed. Additionally, genes encoding L-type HVA Cav channels and LVA Cav channels show slight down-regulation in PFC, while SK channel genes are not differentially expressed across regions. This may help explain why dAdap is not significantly different between PFC and V1. However, due to the lack of statistical significance, this interpretation remains tentative and would benefit from further validation, ideally using a patch-seq dataset from macaque PFC.

Fold-change comparisons between cell types in V1 and PFC are shown in Figure S11D.

### A Hodgkin-Huxley model produces testable predictions

To further illustrate the mechanisms of SRA and generate testable predictions, we construct a Hodgkin-Huxley model that incorporates LVA (T-type) and HVA (L-type) Cav channels, as well as SK channels (Figure 7A). The model also includes intracellular calcium dynamics, where the increase in (Ca^2+^) is proportional to the Ca^2+^ current through HVA Cav channels (*I*_Ca-L_), but not LVA Ca channels, consistent with previous findings^23^^,^^41^ (see STAR Methods).

**Figure 7:**
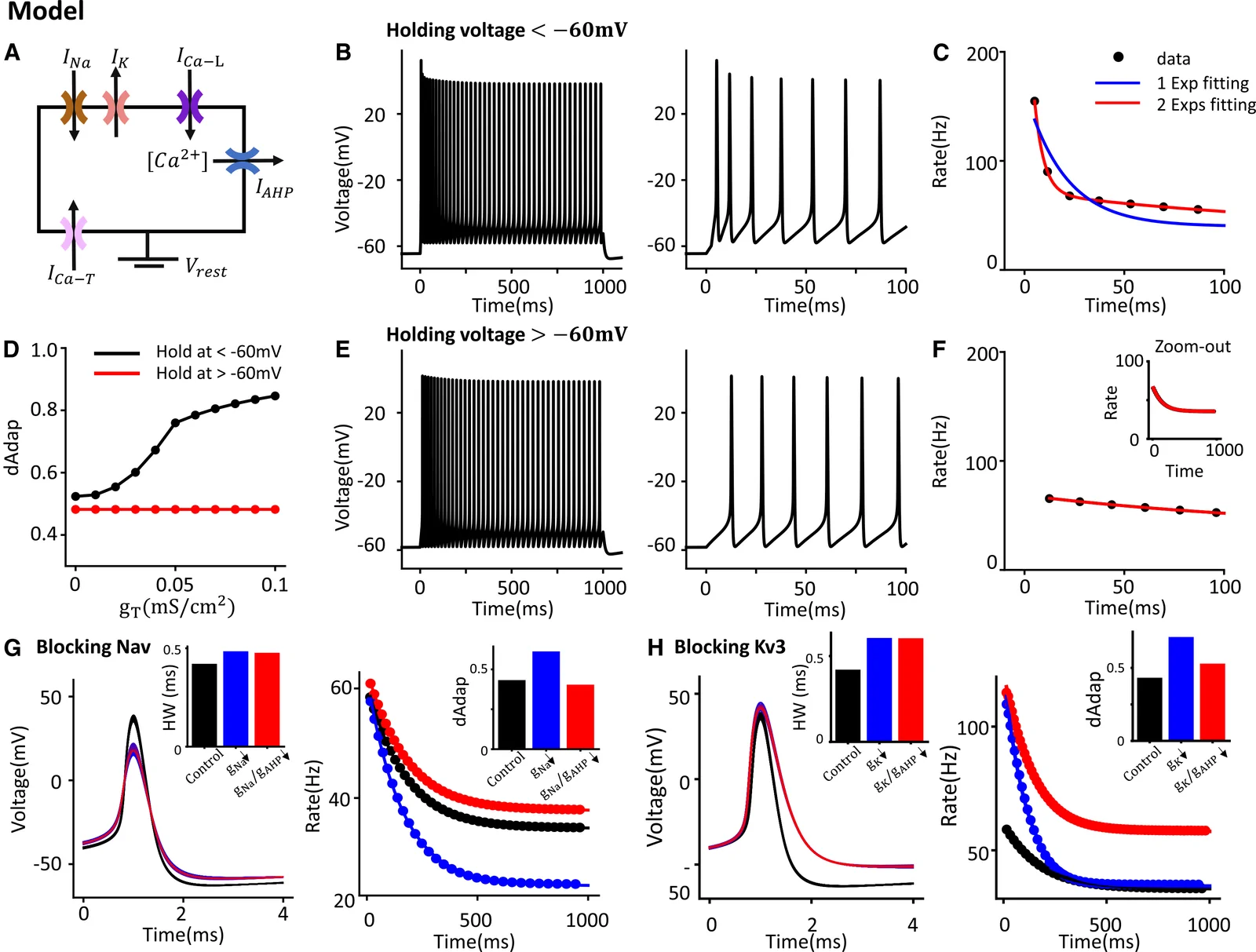
A Hodgkin-Huxley model produces testable predictions (A) Schematic of the model, which includes five ion channels. Intracellular Ca^2+^ concentration is modeled as proportional to *I*_Ca-L_. (B) Example voltage trace from the model when the holding voltage is below −60 mV. Right, zoom-in of the first 100 ms. (C) Instantaneous firing (IF) curve fitted with one versus two exponential components. The two-component fit provides a better match. (D) Model-derived dAdap values under different LVA Ca channel conductances (*g*_*T*_), with holding voltage below −60 mV (black) or above −60 mV (red). At high holding voltages, the T-type channel is inactivated before the depolarizing current. (E and F) Same as (B and C), but with holding voltage above −60 mV. In (F), the one- and two-component fits are identical, indicating the absence of fast SRA. The trace from 1 to 1000 ms is shown as an inset. (G) Predicted changes in HW and dAdap following Nav channel blockade. Three conditions are shown: control ([*g*_*K*_, *g*_*Na*_, *g*_*AHP*_] = [15, 35, 3] ms/cm^2^), reduced *g*_*Na*_ ([15, 25, 3] ms/cm^2^), and reduced *g*_*Na*_ and *g*_*AHP*_ ([15, 25, 2] ms/cm^2^). Insets show HW = [0.42, 0.48, 0.48] and dAdap = [0.43, 0.61, 0.40] for the three conditions, respectively. (H) Predicted changes in HW and dAdap following Kv3 channel blockade. AP waveform and firing-rate adaptation are shown for the control condition ([*g*_*K*_, *g*_*Na*_, *g*_*AHP*_] = [15, 35, 3] ms/cm^2^), reduced *g*_*K*_ ([5, 35, 3] ms/cm^2^), and reduced *g*_*K*_ and *g*_*AHP*_ ([5, 35, 2] ms/cm^2^). The insets show HW and dAdap for these three conditions. HW values are (0.42, 0.61, 0.60), respectively; dAdap values are (0.43, 0.71, 0.53), respectively.

Our model first predicts that the presence of fast timescale SRA depends on the holding voltage. We successfully reproduce SRA with both fast and slow components (Figures 7B and 7C), as demonstrated by fitting the IF curve with one or two exponential decay components (Figure 7D), recapitulating the behavior observed in adaptive SST cells (Figure 2K). Increasing the LVA Ca channel conductance (*g*_*T*_) enhances dAdap (Figure 7E, black line).

Because fast SRA depends on activation of LVA Ca channels, holding the membrane potential above −60 mV prior to depolarization should eliminate this component. Consistent with that, depolarizing the holding voltage removed the OB feature in VIP INs,^48^ and resulted in a smaller after depolarization or delayed depolarization in human neocortical neurons.^49^ In our model, when the model neuron is held just above −60 mV, T-type channels become fully inactivated before depolarization, resulting in a reduced onset firing rate (Figures 7E and 7F). Fitting the IF curve with one or two exponential components yields identical results (Figure 7H), indicating the absence of fast timescale SRA. In this condition, dAdap becomes independent of LVA Ca channel conductance (*g*_*T*_) (Figure 7D, red).

Second, for the slow timescale adaptation, we predict that the mechanisms modulating the HW will also modulate dAdap, but the mechanisms modulating the dAdap may not modulate HW. To show that in the simulation, we first eliminate the fast component by setting the LVA Ca channel conductance (*g*_*T*_) to zero. We then reduce the Nav conductance (*g*_*Na*_) and sequentially reduce the SK conductance (*g*_*AHP*_) (Figure 7G). Reducing *g*_*Na*_ reduces the depolarizing current, broadening the AP waveform, and increasing HW. The wider AP allows greater Ca^2+^ influx during each spike, resulting in increased dAdap (Figure 7G, blue). Further reducing *g*_*AHP*_ selectively decreases the SK current without altering the AP waveform. As a result, dAdap decreases while HW remains unchanged (Figure 7G, red). This prediction can be tested experimentally by sequentially blocking Nav channels with tetrodotoxin (TTX) and SK channels with apamin. In addition, our model also predicts that reducing Kv3 conductance will produce a similar effect (Figure 7H), which can be tested experimentally by blocking Kv3 channels with tetraethylammonium (TEA).

Finally, we predict that blocking upstream L-type Ca^2+^ channels with toxins such as nimodipine affects dAdap but not HW. To test this, we systematically vary the conductance of Kv3 (*g*_*K*_) and HVA Ca^2+^ channels (*g*_*Ca*−*L*_) (Figure S12). HW is primarily determined by *g*_*K*_ and remains unaffected by changes in *g*_*Ca*−*L*_ (Figure S12A), whereas dAdap increases either when *g*_*K*_ is reduced or *g*_*Ca*−*L*_ is increased (Figure S12B). This dissociation demonstrates that Ca^2+^ influx through L-type channels acts upstream of SK channels to regulate SRA.

## Discussion

Our analysis links transcriptomic differences to AP shape and SRA across subtypes, regions, and species. In mouse V1, HW differences correlate with Kv3 and Nav gene expression, consistent with prior studies. dAdap differences are not explained by SK channels, but rather by upstream Ca^2+^ influx, which is shaped by HW and HVA Cav channel-related genes. Additionally, dAdap is influenced by the OB feature, which is modulated by LVA Cav conductance. Using an unsupervised filtering approach, we identify additional contributing genes. Similar relationships are observed in mouse M1 and macaque datasets. Finally, we build a minimal Hodgkin-Huxley model with Ca dynamics to generate testable pharmacological predictions.

Our work pioneers the use of new transcriptomic datasets to understand important electrophysiological features of neurons across regions and species. Our methodology becomes available only because of improvements in sequencing technologies. So far, the rich transcriptomic datasets are mostly used in distinguishing different cell types and developing downstream gene-editing tools.^3^^,^^25^^,^^28^^,^^37^ However, in many scenarios, this line of study is difficult to unravel the intricate functions of individual genes in explaining neural electrophysiological activities. Early work from Tripathy et al.^50^ identifies potential genes by combining transcriptomic datasets with electrophysiological datasets. Patch-seq provides electrophysiological and transcriptomic data from the same cell, providing a unique link at an unprecedented level.^5^ Only by utilizing these data in the patch-seq can we infer the causal links between gene expression differences and HW and dAdap differences, which can be further tested in other datasets across brain areas and species. We study the SRA because of its important role in many functions, including sensory processing from vision,^10^ audition,^11^ olfaction,^12^ and many others.^13^^,^^14^^,^^16^^,^^17^^,^^18^ Based on our analysis, we can test whether the adaptation differences of INs across brain areas lead to functional differences between sensory areas and prefrontal areas in a model. Further, we can infer the strength of IN adaptation across the brain areas based on the multi-regional transcriptomic database and test their functional role in a large-scale model, similar to.^51^^,^^52^ Naturally, our methodology is not limited to the SRA but can be applied to understand other important features across brain areas, such as bursting, sub-threshold oscillation, etc.

When analyzing SRA, the most intuitive hypothesis is that dAdap differences arise from variations in SK channel conductance between PV and SST INs, given that SK channels mediate the mAHP underlying SRA.^22^ However, we find no positive correlation between SK channel-encoding genes and dAdap (Figure 2E). If SK channels were considered in isolation, one might mistakenly conclude they do not contribute to SRA in INs. This highlights the importance of interpreting gene function within the full physiological context, rather than drawing conclusions from a single molecular component.

Further analysis reveals that SK channel activation depends on two upstream factors: the time window and the flow rate of Ca^2+^ influx via HVA Cav channels. Since SST INs have broader APs than PV cells (i.e., larger HW, Figure 1A), they allow more Ca^2+^ influx per AP. Also, the HVA Cav encoding genes *Cacna1b*, *Cacna1c*, *Cacna1e* are more expressed in SST INs (Figure 3), suggesting a higher Ca^2+^ flow rate during APs for SST INs. Additionally, we find that *Cacna1g*, encoding an LVA T-type Ca channel, contributes independently to SRA via its fast inactivation following depolarization above −60 mV.^53^

In our model, we assume that Ca^2+^ influx through LVA T-type Ca channels does not contribute to SK channel activation. However, a study in rat pyramidal cells^41^ suggests that LVA T-type Ca channels may contribute to the mAHP, albeit to a lesser extent than other Ca channel types. Additionally, *Cacna1e*, which encodes R-type Ca channels, is significantly correlated with dAdap, even though the same study reported no role for R-type channels in mAHP generation. It’s worth noting that their experimental protocol was limited to evoking mAHP with a single AP over a 50 m s window, which may not fully capture the dynamics relevant to SRA measured over a 1 s stimulus train. In addition, the spatial distribution of Ca channels may differ between pyramidal cells and INs, potentially affecting their contribution to SRA. Moreover, the study did not isolate the net effect of T-type channel inactivation, leaving its role unresolved. At last, we cannot fully rule out the possibility that *Cacna1e* and *Cacna1g* may be more of a marker gene in distinguishing PV and SST INs. Further research is needed to clarify the distinct contributions of various Ca channel types to SRA under more physiologically relevant conditions.

Our unsupervised gene analysis revealed additional genes involved in intracellular Ca^2+^ modulation. Among them, *Ryr3* (ryanodine receptor 3) is significantly correlated with dAdap but not HW (Figure 4C), consistent with its known role in mediating Ca^2+^ release from intracellular stores.^22^^,^^23^ A recent modeling study^54^ also implicates *Ryr3* in adaptation changes in neuropathic pain, further supporting its potential contribution to dAdap regulation. Other mechanisms may involve the M-type K^+^ current,^24^ which activates rapidly during depolarization and deactivates slowly below threshold. However, *Kcnq5*, a gene encoding this current, is negatively correlated with dAdap (Figure S8C), suggesting it may not account for local dAdap differences. Nonetheless, it may contribute to regional differences, as pyramidal cells in PFC exhibit both higher *KCNQ5* expression and greater dAdap compared to those in V1 (Figure 6E).

H-type K^+^ currents have also been linked to mAHP,^24^ but likely do not impact SRA in our recordings, as cells are depolarized near threshold during the 1 s stimulus. Interestingly, *Hcn1*, which encodes the H-current, is highly expressed in PV cells (Figure 4B), contradicting prior findings that sag currents—primarily mediated by H-currents—are smaller in PV than SST cells in both mouse visual cortex^55^ and human neocortex.^56^ This discrepancy may reflect cell-type specific differences in the kinetics and voltage dependence of *I*_*h*_ in fast-spiking interneurons.^57^

Finally, we emphasize the importance of handling patch-seq data with care. On the electrophysiology side, datasets often adopt uniform recording protocols to enable large-scale sampling across brain regions. However, the high variability in cellular responses can obscure key features. For instance, we found that dAdap measurements are only robust when recordings reach at least 1.5× rheobase (Figure 1A). Since PV INs have much higher rheobase values than SST INs, a fixed maximum current injection (e.g., 500 pA) may be sufficient for SSTs but inadequate for PVs, introducing sampling bias in electrophysiological feature statistics.

On the transcriptomic side, careful noise control is essential for meaningful interpretation. Because our analysis focuses on a small number of genes involved in the hypothesized mechanisms, we cannot rely on the statistical power afforded by large gene sets typically used in classification studies. To mitigate this, we apply stringent thresholds for total gene counts and feature detection, exclude potentially contaminated cells, and analyze data at the transcriptomic subtype (T-type) level. Additionally, differences between and within cell classes may arise from distinct underlying mechanisms.^7^ *Kcnc1*, for example, shows a significant class-dependent effect, indicating divergent regulatory logic in excitatory neurons versus interneurons.

In summary, we show that transcriptomic data can account for differences in AP shape and SRA across cell types and brain regions. Our analysis suggests that expression differences in both HVA and LVA Ca channel-encoding genes, along with HW-related genes, rather than SK channel genes, underlie SRA variability. This underscores the importance of avoiding conclusions based on single-gene analyses. Our methodology offers a framework for predicting electrophysiological properties across brain regions using existing transcriptomic data, paving the way for future experimental and theoretical studies.

### Limitations of the study

The present study has several limitations. First, the transcriptomic-electrophysiological relationships identified here are correlational and lack direct experimental validation. Our computational model generates falsifiable experimental predictions, but these predictions remain to be tested. Second, although the proposed mechanisms generalize across PV, SST, VIP, and SNCG interneurons, they do not explain the electrophysiological properties of LAMP5 interneurons, suggesting that additional mechanisms contribute to SRA in this population. Third, the macaque analysis is limited by the relatively small electrophysiological dataset and by the use of separate electrophysiological and transcriptomic datasets, which precludes direct cell-level validation of the proposed relationships.

## Resource availability

### Lead contact

Requests for further information and resources should be directed to and will be fulfilled by the lead contact, Xiao-Jing Wang (xjwang@nyu.edu).

### Materials availability

This study did not generate new materials.

### Data and code availability

• This paper analyzes existing, publicly available patch-seq, single-cell RNA-seq, and transcriptomic datasets. Accession numbers and links to these datasets are listed in the key resources table. Processed data and statistical analyses supporting the figures are included in the Tables S1, S2, S3, S4, S5, S6, and S7.
• All original code has been deposited on GitHub at https://github.com/johnhongyumeng/Patchseq and is publicly available as of the date of publication.
• Any additional information required to reanalyze the data reported in this paper is available from the lead contact upon request.

## Acknowledgments

We thank Zhenghan Liao, Panagiota Theodoni, Loïc Magrou, and other members of X.-J.W. lab for the discussion. We thank Shlomo Dellal, Robert Machold, Chiung-Yin Chung, and other members of B.R. lab for the discussion. We thank Shreejoy Tripath for his suggestions and feedback on this work. We thank Julia Sunstrum, Sam Mester, Meghan Wiederman, Sara Matovic, Michelle Jimenez, and Michael Poulter for the electrophysiological data collection of the macaque monkeys. We thank Maximiliano José Nigro for sharing mouse S1 L23 data. We thank Yidi Sun and Juan Meng from ION, Shanghai, for sharing the transcriptomic data of the macaque monkey and for their help with the technique. This work is supported by 10.13039/100000002NIH grant R01MH062349, ONR grant N00014-23-1-2040, and the James Simons Foundation grant NC-GB-CULM-00003138 (to X.-J.W.), Neuronex NSF 2015276 (to Amy Arnsten and X.-J.W.), 10.13039/100000002NIH grants P01NS074972 and R01NS133751 (to B.R.), 10.13039/501100000024CIHR, 10.13039/501100000038NSERC, Autism Research Chair province of Ontario, 10.13039/100031042NeuroNex (to J.M.-T.), the Canadian Institute for Health Research (NGN 171424) in relationship to Neuronex NSF 2015276 and BrainsCAN accelerator grant funded by a Canada First Research Excellence Fund (to W.I.).

## Author contributions

Conceptualization, J.H.M., B.R., and X.-J.W.; methodology, J.H.M., Y.K., W.I., J.M.-T., B.R., and X.-J.W.; investigation, J.H.M., Y.K., A.L., and M.F.; writing – original draft, J.H.M. and Y.K.; writing – review and editing, J.H.M, Y.K., B.R., and X.-J.W.; funding acquisition, W.I., J.M.T., B.R., and X.-J.W.; supervision, J.M.T., B.R., and X.-J.W.

## Declaration of interests

The authors declare no competing interests.

## Declaration of generative AI and AI-assisted technologies in the manuscript preparation process

During the preparation of this work, the authors used ChatGPT (OpenAI) to assist with proofreading and improving the clarity of the manuscript. After using this tool, the authors reviewed and edited the content as needed and take full responsibility for the content of the publication.

## STAR★Methods

### Key resources table

**Table:** 

REAGENT or RESOURCE	SOURCE	IDENTIFIER
**Deposited data**		

Mouse V1 Patch-seq dataset	Allen Institute for Brain Science; Gouwens et al.^25^	https://knowledge.brain-map.org/data/1HEYEW7GMUKWIQW37BO/summary; DANDI:000020;
Mouse M1 Patch-seq dataset	Scala et al.^26^	https://github.com/berenslab/mini-atlas; DANDI:000008
Mouse dissociated single-cell RNA-seq reference dataset	Yao et al.^28^	NeMO: nemo:dat-jb2f34y; GEO: GSE185862
Macaque cortex transcriptomic dataset	Chen et al.^3^	CNGB: CNP0002035; https://macaque.digital-brain.cn/spatial-omics

**Software and algorithms**		

Patch-seq data analysis workflow	This paper	https://github.com/johnhongyumeng/Patchseq
Python 3.6 (Allen data analysis)	Python Software Foundation	https://www.python.org
Python 3.13 (model simulation)	Python Software Foundation	https://www.python.org
Brian2 2.9.0 (model simulation)	Brian Simulator Developers	https://brian2.readthedocs.io
MATLAB R2024b	MathWorks	https://www.mathworks.com/products/matlab.html
R 4.2.3	R Core Team	https://www.r-project.org
SciPy (curve_fit)	SciPy Developers	https://scipy.org
lmfit	lmfit Developers	https://lmfit.github.io/lmfit-py/
DESeq2 1.36.0	Love et al.^58^; Bioconductor	https://bioconductor.org/packages/DESeq2/
clusterProfiler 4.4.4	Yu et al.^59^; Wu et al.^60^	https://bioconductor.org/packages/clusterProfiler/

**Other**		

Mouse S1 layer 2/3 electrophysiology dataset	He et al.^61^; Nigro et al.^62^	N/A
Macaque intracellular electrophysiology dataset	Inoue and Martinez labs; Jimenez thesis	N/A

### Experimental model and study participant details

No new animal experiments were performed in this study; all analyses used previously collected datasets.

The transcriptomic and electrophysiological data were accessed from the Allen Institute for Brain Science Cell Types Database - Mouse Patch-seq dataset.^25^ The original study used male and female mice aged P45–P70 on a C57BL/6 J background, with animal procedures approved by the Institutional Animal Care and Use Committee of the Allen Institute for Brain Science. Electrophysiological recordings were made at physiological temperature (34 °C). This dataset contains 4,284 mouse cells from the primary visual cortex (VISp) layers 1, 2/3, 4, 5, 6a, and 6 b. Cells tagged with PV, SST, VIP, SNCG, and LAMP5 are included in the analysis.

The Patch-seq dataset of adult mouse primary motor cortex (MOp) was accessed from.^26^ The original study used male and female mice aged P35–P245 on a C57BL/6 background, with animal procedures approved by the Institutional Animal Care and Use Committee of Baylor College of Medicine. This dataset contained transcriptomic and electrophysiological data from 1,329 cells recorded at room temperature (25 °C) from layers 1, 2/3, 5, and 6 of mouse MOp. Cells tagged as PV, SST, and pyramidal (ET, IT, CT, and NP) cell types are included in our analysis.

The electrophysiological dataset from mouse S1 layer 2/3 was collected from PV-Cre-Ai9 or SST-Cre-Ai9 lines as in.^61^ Tissue acquisition, processing, and solutions followed the procedures described by Nigro et al.^62^; animal procedures followed NIH guidelines and were approved by the Institutional Animal Care and Use Committee of NYU School of Medicine. The dataset analyzed here was restricted to layer 2/3 and consisted of 16 PV cells and 21 SST cells from 20 individuals of either sex within an age range of 18–35 days.

The macaque transcriptomic dataset, collected through unique molecular identifier (UMI) sequencing, was accessed from.^3^ We used data from monkey No. 2, a 4-year-old male cynomolgus macaque (*Macaca fascicularis*). Animal procedures were approved by the Biomedical Research Ethics Committee of the CAS Center for Excellence in Brain Science and Intelligence Technology. We excluded data with ambiguous area annotation, i.e., data from slices across two or more areas. Data from all layers were used.

The intracellular recordings of macaque neurons were obtained in the Inoue and Martinez-Trujillo laboratories. The dataset included rhesus macaques (*Macaca mulatta*) and cynomolgus macaques (*Macaca fascicularis*). Tissue acquisition, processing, and recording procedures are described by Jimenez-Sosa.^63^ The dataset consisted of 247 recorded neurons from 11 individuals of both sexes, with an age range of 4.39–14.6 years. Animal experiments were conducted at Western University in accordance with Canadian Council on Animal Care guidelines and with approval from the University Animal Care Committee.

Sex was not included as an independent variable in the present analyses; therefore, potential sex-dependent effects were not assessed and remain a limitation of the study.

### Method details

#### Electrophysiological measurements

We used the same measurement as in.^36^ We automatically defined a detection threshold to detect an action potential (AP) in any given voltage trace *V*(*t*) by the following: We first defined an upper bound as *V*_*up*_ = min{max(*V*(*t*)), 0}, then a lower bound as *V*_*low*_ = median(*V*(*t*)). Further, we set the detection threshold as *V*_*thr*_ = max{0.8*V*_*up*_ + 0.2*V*_*low*_, −20}. Next, the *i*th AP is detected at *t*_*i*_ if *V*(*t*_*i*_) < *V*_*thr*_ and *V*(*t*_*i*_ + Δ*t*) ≥ *V*_*thr*_, with the resolution in our recordings Δ*t* = 0.05 *m s*. Furthermore, we exclude any APs occurring within 0.5 *m s* following another AP. The rheobase current is the minimum current that triggers an AP in the experiments.

The AP-related properties are calculated from aggregated APs from all sweeps with fewer than 40 APs, while excluding the first AP in each sweep. The maximum voltage of each AP is calculated from the 2 *m s* time window following the trace past the AP detection threshold. The AP threshold is calculated as the voltage when the voltage derivative is 20 *mV*/*ms*. The AP half-width is calculated as the duration for which the AP remained above the midpoint between the peak and the threshold. To improve precision, linear interpolation is used to calculate the threshold and AP half-width.

The degree of adaptation (dAdap) is calculated from the instantaneous firing (IF) curve, excluding the first AP from each recording sweep. IF curves are calculated as *r*(*t*_*i*_) = 1/(*t*_*i*+1_ − *t*_*i*_). Next, we fit the IF curve to an exponential function *f*(*x*) = *a* + *b*exp(−*cx*), using the *curve fit* function of *scipy* package in Python. The timescale of the fitting is required to be larger than 20 m s (equivalently *c* < = 50*Hz*). The dAdap is the proportion of the change in firing rate between 0s and 1s: dAdap = 1 − *f*(1*s*)/*f*(0). Due to the large variance of dAdap around the rheobase, we choose the dAdap from the first sweep with a current injection above 1.5 times the rheobase. In addition, the qualified sweep must be recorded below the four times rheobase, the last spike must occur later than 0.8 s, and the sweep must have at least 10 APs. Otherwise, the cell was excluded from further analysis.

To quantify whether a cell has an onset bursting (OB) feature, we fit the IF curves to a function with two exponential components *f*(*x*) = *a* + *b*_1_exp(−*c*_1_*x*) + *b*_2_exp(−*c*_2_*x*). During the fitting, the timescale of the fast component is required to be less than 20 *m s* (or equivalently *c*_1_ > 50 *Hz*) and the slow timescale is required to be larger than 20 m s (equivalently *c*_2_ < = 50*Hz*). The fast amplitude *b*_1_ is referred to as the rectified onset bursting rate. The significance of OB for a sweep is determined by the *p*-value of the fast amplitude *b*_1_ by the following equation *p* = 2(1 − *t*_cdf_(|*b*_1_.*μ*|/*b*_1_.*σ*), *df*) where *t*_cdf_ is the cumulative probability function of Student’s t-distribution. *b*_1_.*μm* and *b*_1_.*σ* are the mean and standard deviation of fitted parameter *b*_1_, *df* is the degrees of freedom. The fitting is done using the Python package lmfit.

The significance of a cell is determined by the minimum adjusted *p*-value of all the recorded sweeps by the number of sweeps through the BH correction. The corresponding OB rate *b*_1_ is considered the OB rate for the cell. A cell is considered to have the OB feature if the q-value is less than 0.05 and the rectified OB rate *b*_1_ is larger than 10*Hz*. The OB abundance is determined by the number of cells showing a significant q-value *q* < 0.05 that is adjusted by the cell number of the corresponding T-type.

We investigate the effect by removing cells with an OB feature. To do that, we either remove the cells that have an OB feature, or remove the T-types with OB abundance larger than 20%. See Table S1 for details.

#### Computational modeling

We build a Hodgkin-Huxley neuron model equipped with *Ca*^2+^ dynamics, low-threshold and high-threshold voltage-gated *Ca*^2+^ channels corresponding to T-type and L-type calcium channels, respectively, and *Ca*^2+^-activated *K*^+^ ion channels to reproduce the observed heterogeneity spike-width and degree of adaptation in interneurons. The equations are the following:

$$\begin{array}{cc} C_{m}\frac{dV}{dt} & =I_{Na}+I_{K}+I_{L}+I_{\mathrm{CaL}}+I_{\mathrm{CaT}}+I_{\mathrm{AHP}}+I_{\mathrm{inj}}\\ \frac{d\left[Ca^{2+}\right]}{dt} & =-\alpha_{Ca}I_{\mathrm{CaL}}-\frac{\left[Ca^{2+}\right]}{\tau_{Ca}}\\ I_{Na} & =-g_{Na}m_{\infty }^{3}h\left(V-V_{Na}\right)\\ I_{K} & =-g_{K}n^{4}\left(V-V_{K}\right)\\ I_{\mathrm{CaL}} & =-g_{\mathrm{CaL}}m_{\mathrm{CaL}}\left(V-V_{Ca}\right)\\ I_{\mathrm{CaT}} & =-g_{\mathrm{CaT}}m_{T}h_{T}\left(V-V_{Ca}\right)\\ I_{\mathrm{AHP}} & =-g_{\mathrm{AHP}}\left(\frac{\left[Ca^{2+}\right]}{\left[Ca^{2+}\right]+K_{d}}\right)\left(V-V_{K}\right)\\ I_{L} & =-g_{L}\left(V-V_{L}\right) \end{array}$$

Within the equations, *g*_*Na*_,*g*_*K*_,*g*_*CaT*_, *g*_*CaL*_, *g*_*AHP*_ and *g*_*L*_ are the conductance for the voltage-gated sodium, potassium, low-threshold calcium, high-threshold calcium channels, and the calcium-activated potassium channels, respectively; *V*_*Na*_,*V*_*K*_,*V*_*Ca*_, and *V*_*L*_ are the reversal potentials for sodium, potassium, calcium, and the leak currents, respectively.

*I*_*Na*_ and *I*_*K*_ are adapted from,^64^ with all voltage dependencies shifted by +10 mV to match the experimentally observed reset voltage. This adjustment preserves the original AP dynamics while aligning the model more closely with our data.

The gating variables *m*, *h* and *n* satisfy the first-order kinetics given by:

$$\frac{dx}{dt}=\phi_{x}\left[\alpha_{x}\left(V\right)\left(1-x\right)-\beta_{x}\left(V\right)x\right]=\frac{\phi_{x}\left[x_{\infty }\left(V\right)-x\right]}{\tau_{x}\left(V\right)}$$

Here, *ϕ*_*x*_ = 5 is a temperature factor. For *I*_*Na*_, $m_{\infty }=\frac{\alpha_{m}}{\alpha_{m}+\beta_{m}}$ where $\alpha_{m}=-0.1\frac{V+25}{\exp\left(-0.1\left(V+25\right)\right)-1}$ and $\beta_{m}=4\exp\left(\frac{-\left(V+50\right)}{18}\right)$. $\alpha_{h}=0.07\exp\left(\frac{-\left(V+48\right)}{20}\right)$ and $\beta_{h}=\frac{1}{\exp\left(-0.1\left(V+18\right)\right)+1}$. For *I*_*K*_, $\alpha_{n}=\frac{-0.01\left(V+24\right)}{\exp\left(-0.1\left(V+24\right)\right)-1}$ and $\beta_{n}=0.125\exp\left(\frac{-\left(V+34\right)}{80}\right)$.

We adopt the low-threshold *Ca*^2+^ dynamics from,^65^ where *m*_*T*,*∞*_ = *H*(*V* − *V*_*h*_) where *H* is the Heaviside step function and *V*_*h*_ is the threshold responsible for the activation of burst spiking. The inactivation variable *h*_*T*_ has dynamics given by:

(Equation 1) $$\frac{dh_{T}}{dt}=\left\{\begin{array}{cc} -h_{T}/\tau_{h^{-}} & \left(V>V_{h}\right)\\ \left(1-h_{T}\right)/\tau_{h^{+}} & \left(V<V_{h}\right) \end{array}\right)$$

We then adopted the high-threshold *Ca*^2+^ dynamics and adaptation current *I*_*AHP*_ through the *Ca*^2+^ activated SK channel from,^66^ where. $m_{\infty }\left(V\right)=\frac{1}{1+\frac{\exp\left(-\left(V+20\right)\right)}{9}}$

We use the following parameter values unless otherwise specified: *C*_*m*_ = 1 *μ*F/cm^2^, *g*_*L*_ = 0.1 mS/cm^2^, *g*_*Na*_ = 35 mS/cm^2^, *g*_*K*_ = 9 mS/cm^2^, *g*_*CaL*_ = 0.1 mS/cm^2^, *g*_*CaT*_ = 0.05 mS/cm^2^, *g*_*AHP*_ = 3 mS/cm^2^, *K*_*d*_ = 30 *μ*M, $\tau_{h^{-}}=5ms$, $\tau_{h^{+}}=100ms$, *τ*_*Ca*_ = 200 m s, *α*_*Ca*_ = 0.002 *μ*M ⋅ *cm*^2^/(ms ⋅ *μ*A), and reversal potentials *V*_*L*_ = −55 mV, *V*_*Na*_ = 65 mV, *V*_*K*_ = −80 mV, *V*_*Ca*_ = 120 mV, with *V*_*h*_ = −60 mV as the T-type channel threshold.

The dAdap is always measured from the sweep with a depolarization current injection of *I*_inj_ = 2 *μ*A/cm^2^. For low holding potentials, the default holding current is $I_{\mathrm{inj}}^{\text{hold}<-60mV}=-1\mu A/cm^{2}$. To test conditions under which the T-type Ca channel is inactivated, when the holding voltage is high, the holding current is raised to $I_{\mathrm{inj}}^{\text{hold}>-60mV}=-0.4\mu A/cm^{2}$.

#### Quality control of the transcriptomic data

From the raw counts in the Patch-seq database, we exclude cells whose number of unique sequenced genes is less than 6600 and cells whose total counts are less than 6 × 10^5^ (Figure 2B, Figure S2A). Genes detected in fewer than 100 cells were filtered out. Raw counts were normalized by count per million (CPM) and then transformed to log_2_(*CPM* + 1). We refer to a dissociated single-cell dataset^28^ as the uncontaminated database. Following,^30^ the contamination score *CS* quantifies the extent of microglial marker-gene expression^67^ in the sequenced cells.

Single-cell RNA samples collected by Patch-seq could be contaminated by neighboring microglial cells, but not those collected by dissociated cells.

$$CS_{N\text{\_}M}=\frac{P_{N\text{\_}M}-D_{N\text{\_}M}}{D_{M\text{\_}M}-D_{N\text{\_}M}}$$

Here, *X*_*A*-*M*_ represents the median of 50 marker genes from the Patch-seq (P) or dissociated (D) dataset of type *A* in microglial cells. The marker genes differ across cell types (PV or SST IN, pyramidal cell, microglial cell). We adopt the marker gene list for each cell type from.^28^ The contamination score *CS* ranges from 0 to 1, reflecting the excess of off-target marker expression. We limited our analysis to cells with *CS* < 0.2 (Figures 2B and 5B).

### Quantification and statistical analysis

Statistical significance in the figures is indicated by ∗*p* < 0.05, ∗∗*p* < 0.01, and ∗∗∗*p* < 0.001, unless otherwise stated.

#### Classifier

To train a classifier based on HW and dAdap, we find the linear decision boundary that best separates the cells by using the support vector machine (SVM) in MATLAB. The resulting decision boundary is defined by *β*_1_*x* + *β*_2_*y* + *b* = 0. To test the robustness of this method, we randomly split the data points into an 80% training set and a 20% testing set. We calculate the average performance of 100 repetitions < Accy_test_ >. To identify cells with low classification accuracy, we employed a Gaussian mixture model (GMM). This model assumes that the data from a cell type are generated from a mixture of two Gaussian distributions, calculated by MATLAB’s *fitgmdist* function. The classification of each data point was based on the highest posterior probability derived from the posterior function, which computes the likelihood that each data point belongs to each Gaussian component. The curves with 95% posterior-probability for each cluster are reported as confidence boundaries.

The fitted parameters are included in Table S1.

#### Multi-gene linear regression model

Some genes, though passing QC in the published dataset, exhibit zero counts in many cells—a common issue known as dropout. This is illustrated in Figures S2D and S2F. A zero count may reflect either truly low expression or a detection failure in a cell that does express the gene. To minimize the impact of this ambiguity, we quantify the expression prevalence for each gene, defined as the number of cells with non-zero counts. We then exclude genes in the bottom 10th percentile of the expression prevalence distribution from subsequent analyses.

Each cell in the dataset is associated with a T-type tag. The mean trimmed expression for each T-type is calculated by averaging the expression of individual cells after removing the top and bottom 25% expression.

We used a weighted linear regression to study the correlation between HW or dAdap with different genes. The *p*-value for a nonzero slope *p* and the variance explained *R*^2^ are reported for each pair. When comparing multiple models, a correction for the multiple testing problem is necessary. To do so, we adjusted *p*-values with the false discovery rate (FDR) control using the Benjamini-Hochberg method^68^ (Figures 3A and 3B; Figure S6).

We train the multiple linear regression model to predict HW or dAdap based on genes. We first test the performance of the prediction by including all the significantly correlated genes identified in Figure 3.

$$\text{HW}\;or\;\text{dAdap}=\sum \alpha_{i}g_{i}+\beta$$

where *g*_*i*_ is the log_2_ of a gene, and *β* is the intercept. The weights *α*_*i*_ are fitted through a multivariable linear regression. Since the subunits encoded by the SK-family can form a heterotetramer, we use a single independent variable for the whole SK family:

$$g_{SK}=\log_{2}\left(\text{CPM}\left(Kcnn1+Kcnn2+Kcnn3\right)+1\right)$$

We also test the type-dependent effect by including a variable when fitting the model to PV and SST INs:

$$g_{\mathrm{type}}=\log_{2}\left(\text{CPM}\left(Sst+1\right)\right)-\log_{2}\left(\text{CPM}\left(Pvalb+1\right)\right)$$

This variable is dropped in the AIC-selected model, suggesting the type-dependent effect, if any, is minimal.

Given the limited number of T-types, the more genes we include, the more likely we are to observe an overfitting problem. We next applied the Akaike Information Criterion (AIC) to select the model that explains the greatest variance with the fewest genes.^69^^,^^70^ The genes and corresponding fitted weights are listed in the Table S3 file.

After multi-linear regression and auto-selection based on AIC, we next calculated partial R^2^. To quantify the unique contribution of specific gene families within the auto-selected multi-regression model, we computed both the partial R^2^ and the adjusted partial R^2^ for each gene family.

Compared to the AIC-selected model, we define reduced models by removing the genes that belong to each gene families. The partial R^2^ was computed as the proportion of residual sum of squares (RSS) reduction when moving from the reduced model to the full model:

$$R_{\text{partial}}^{2}=\frac{RSS_{\text{reduced}}-RSS_{\text{full}}}{RSS_{\text{reduced}}}$$

where *RSS*_reduced_ is the residual sum of squares from the reduced model (excluding the term(s) of interest), and *RSS*_full_ is the residual sum of squares from the full model.

This value reflects the additional variance explained by the removed genes, beyond what is already explained by the remaining genes.

To account for model complexity and sample size, we additionally computed adjusted partial R^2^ using the following formula:

$$R_{\text{partial,}\;\text{adj}}^{2}=1-\left(\frac{\left(1-R_{\text{partial}}^{2}\right)\left(n-k_{\text{full}}-1\right)}{n-k_{\text{reduced}}-1}\right)$$

where *n* is the number of observations (sample size), which was the same for both models, and *k*_*full*_ and *k*_*reduced*_ are the number of estimated coefficients excluding the intercept in the full and reduced models, respectively. This adjusted measure adjusts for differences in model complexity, providing a more conservative estimate of unique explanatory power.

The fitted coefficient of each remaining independent variable is reported in Figure S7. However, given the strong correlation in gene expression (Figure S5B), the exact weight does not necessarily reflect the corresponding channel conductance and should be interpreted with caution.

#### Regression with respect to cell class

To test which genes have a class-driven effect in explaining HW or dAdap across INs versus pyramidal cells, we fit a class-driven linear model as in^7^:

(Equation 2) $$\text{dAdap}\;\text{or}\;\text{HW}=\alpha_{1}g_{i}+\alpha_{2}c_{i}+\alpha_{3}g_{i}c_{i}+\beta$$

where *g*_*i*_ is the log_2_(*CPM* + 1) of a T-type, *c*_*i*_ is a categorical variable that indicates the class for the *i*th T-type, which is *c*_*i*_ = 1 if the T-type is an IN T-type, or *c*_*i*_ = 0 if the T-type is a pyramidal neuron T-type. The significance of the class-driven effect is indicated by the adjusted *p*-value for *α*_3_, calculated by the ANOVA test. To account for this class-dependent effect, we further reported *p*-values for pyramidal cells and interneurons separately by fitting linear models to the pyramidal cell data and the interneuron data, respectively. The details of the analysis are listed in Table S5 file.

#### Differential expression analysis

Differential expression analysis was performed with R 4.2.3 and the DESeq2 package in R (1.36.0)^68^. In analyzing Patch-seq mouse data, genes with q-value <0.05 and log_2_(*foldchange*) > 1 are considered differentially expressed genes (DEGs). In analyzing macaque UMI-sequencing data, genes with q-value <0.01 and log_2_(*foldchange*) > 0.4 are considered differentially expressed genes (DEG). In estimating the size factors of the macaque monkey, we used the “poscounts” option offered by the DESeq2 package to process the data containing many zero counts. Otherwise, we used the default parameters offered by the DESeq2 package.

#### Gene Ontology enrichment

For selected genes, Gene Ontology^46^^,^^47^ enrichment analysis was performed using the R package clusterProfiler (4.4.4) with the over-representation analysis (ORA) approach.^59^^,^^60^

## Supplemental information

Document S1. Figures S1–S12

Table S1. Aggregated V1 data at T-type level, related to Figure 2

Table S2. Parameters for multiple-variable linear regression, related to Figure 3

Table S3. Raw data generated from the unsupervised filtering algorithm, related to Figure 4

Table S4. Raw data generated from V1 GO analysis, related to Figure 4

Table S5. Aggregated M1 data at T-type level, related to Figure 5

Table S6. M1 weighted linear regression results, related to Figure 5

Table S7. DESeq analysis results for macaque data, related to Figure 6
