# Supplementary material for: Patch-seq analysis reveals transcriptomic correlates of neuronal firing rate adaptation across subtypes and species
Source: iScience. 2026 Oct 8;29(11):117712. doi: 10.1016/j.isci.2026.117712 (PMC13676982; doi:10.1016/j.isci.2026.117712)
Supplement: Supplementary file 1 [file mmc1.pdf]

iScience, Volume *29*

Supplemental information

Patch-seq analysis reveals transcriptomic correlates of neuronal firing rate adaptation across subtypes and species

John Hongyu Meng, Yijie Kang, Alan Lai, Michael Feyerabend, Wataru Inoue, Julio Martinez-Trujillo, Bernardo Rudy, and Xiao-Jing Wang

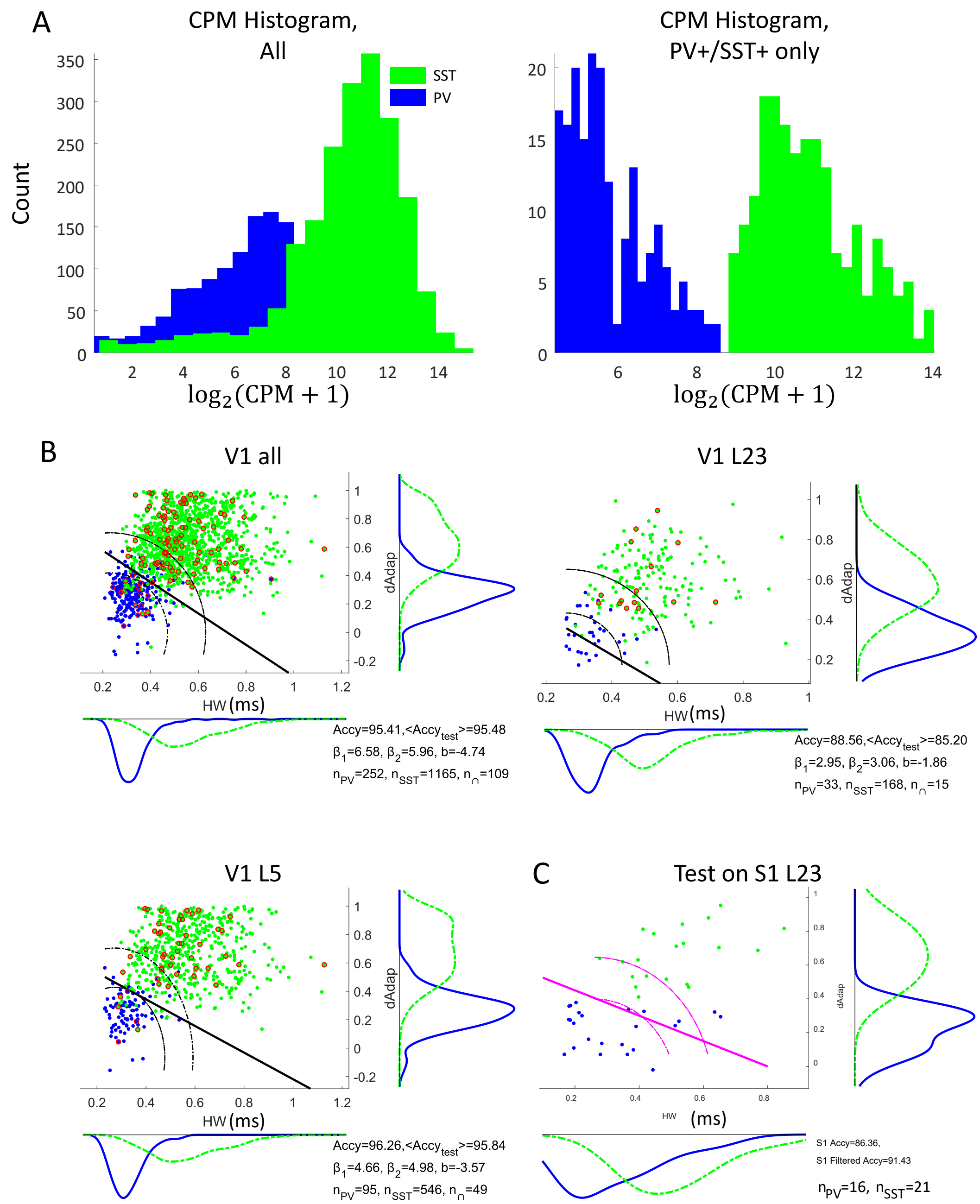

Figure S1: Performance of the trained classifier compared to that of the marker gene classification. Related to Figure 1. (A) distribution of the *Pvalb* and *Sst* markers. Left: Histogram of *Pvalb* and *Sst* in all cells. Right: Histogram of cells that are both *Pvalb*+ and *Sst*+. A cell with *PV* larger than the 20th percentile of the total distribution ($\theta_{PV} = 4.49$) counts as *Pvalb*+. The same applies for *Sst*+ ($\theta_{SST} = 8.97$). In the dataset, a total of 1025 *Pvalb+* cells and 1604 *SST+* cells are identified. Within those, 189 cells are identified as *Pvalb*+/*Sst*+. The CPM distributions of these 189 cells are shown on the right. If considering multiple-gene classification as the ground truth, then the accuracy of the gene-marker (PV and SST marker) classification is 89.62%. (B) Classification of PV and SST based on HW and dAdap across layers. The solid line represents the classification boundary, indicated by $y = -(x\beta_1 + b)/\beta_2$. The red circle represents *Pvalb*+/*Sst*+ cells. Notice that these cells are not only distributed close to the boundary. Dashed line, the 95% confidence boundary based on the Gaussian mixture model. See Spreadsheet S1 for details. (C) Test the trained classifier from V1 on a dataset from the somatosensory cortex. The filtered accuracy represents the classifier accuracy after excluding low-confidence cells. I.e., excluding the cells within the two dashed pink lines.

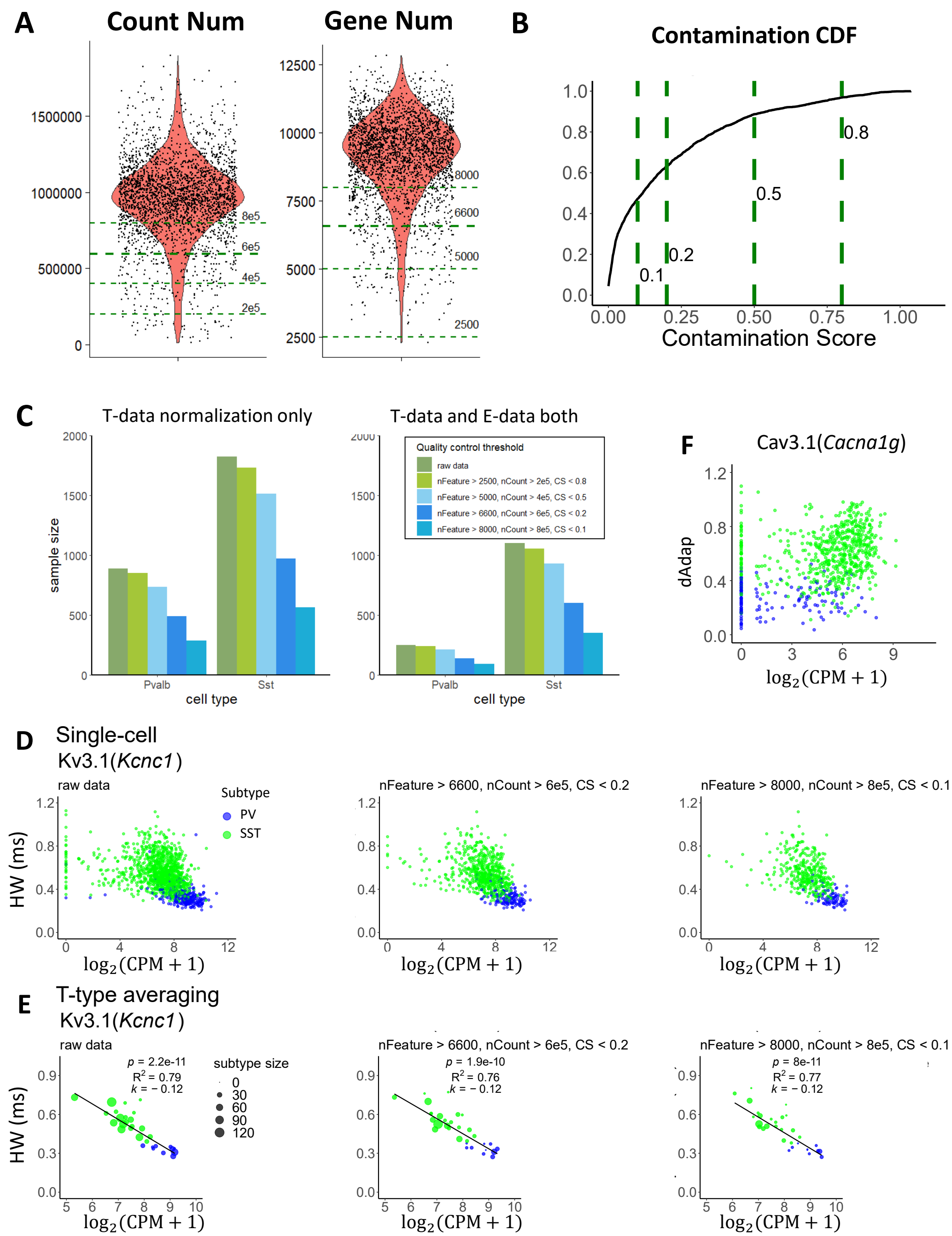

Figure S2: Quality control of transcriptomic data with different thresholds. Related to Figure 2. (A) Violin plot for V1 raw count data: number of total raw counts per cell (nCount_RNA, left) and number of unique sequenced genes per cell (nFeature_RNA, right). Dashed lines represent different thresholds. (B) Cumulative distribution function for the contamination score. (C) Histogram of the number of cells (sample size) that passed the corresponding quality control parameters before (left) and after (right) merging with cells that also passed the electrophysiological quality control. The parameter sets are colored. (D, E) Scatter plot at the single cell level (D) and at the T-type level (E) of the HW and *Kcnc1* with different quality control parameters. Strict quality control reduces dropout issues (zero readouts along the y-axis) at the single-cell level, while T-type level performances are comparable. (F) Scatter plot at the single-cell level of the dAdap and *Cacna1g* after the default quality control. For low-expression genes, the dropout issue remains obvious.

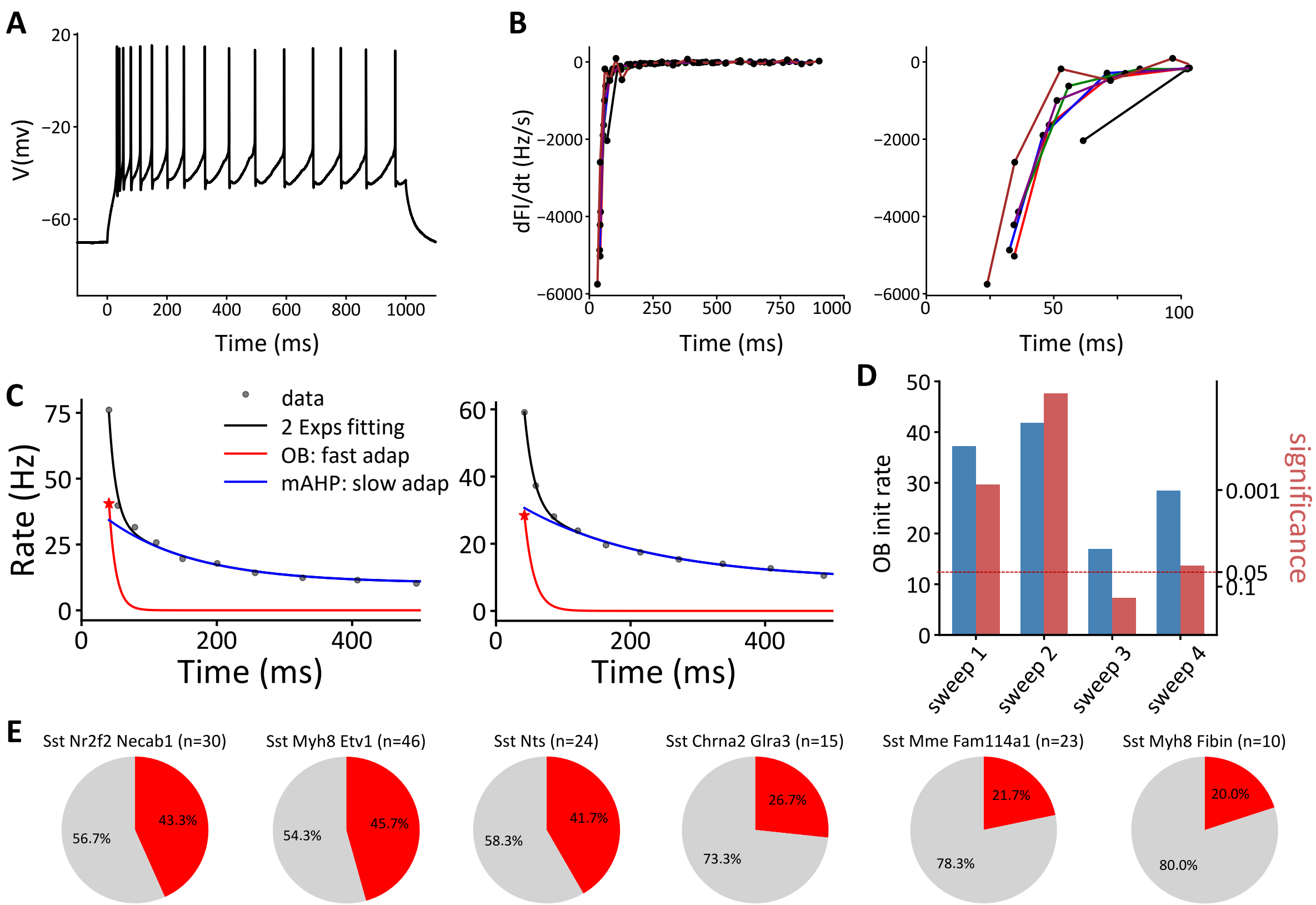

Figure S3: Analysis of the onset bursting feature. Related to Figure 2. (A) Example cell (same as in Figure 2J, K), recorded four times at the same depolarizing current. (B) Instantaneous firing rate (IF) curves across sweeps. Right panel: zoomed-in view. Different colors represent different sweeps. (C) Example IF curve fitted with two exponential components. Color indicates different components of the fitting. Red indicates the fast component of the fitting. (D) Fitted results across the four sweeps. Although three of the four sweeps show a significant OB feature, the initial OB rate varies substantially, making it unsuitable for quantitative analysis. (E) Cells with OB features are enriched in specific T-types. See Table S1 for the full list.

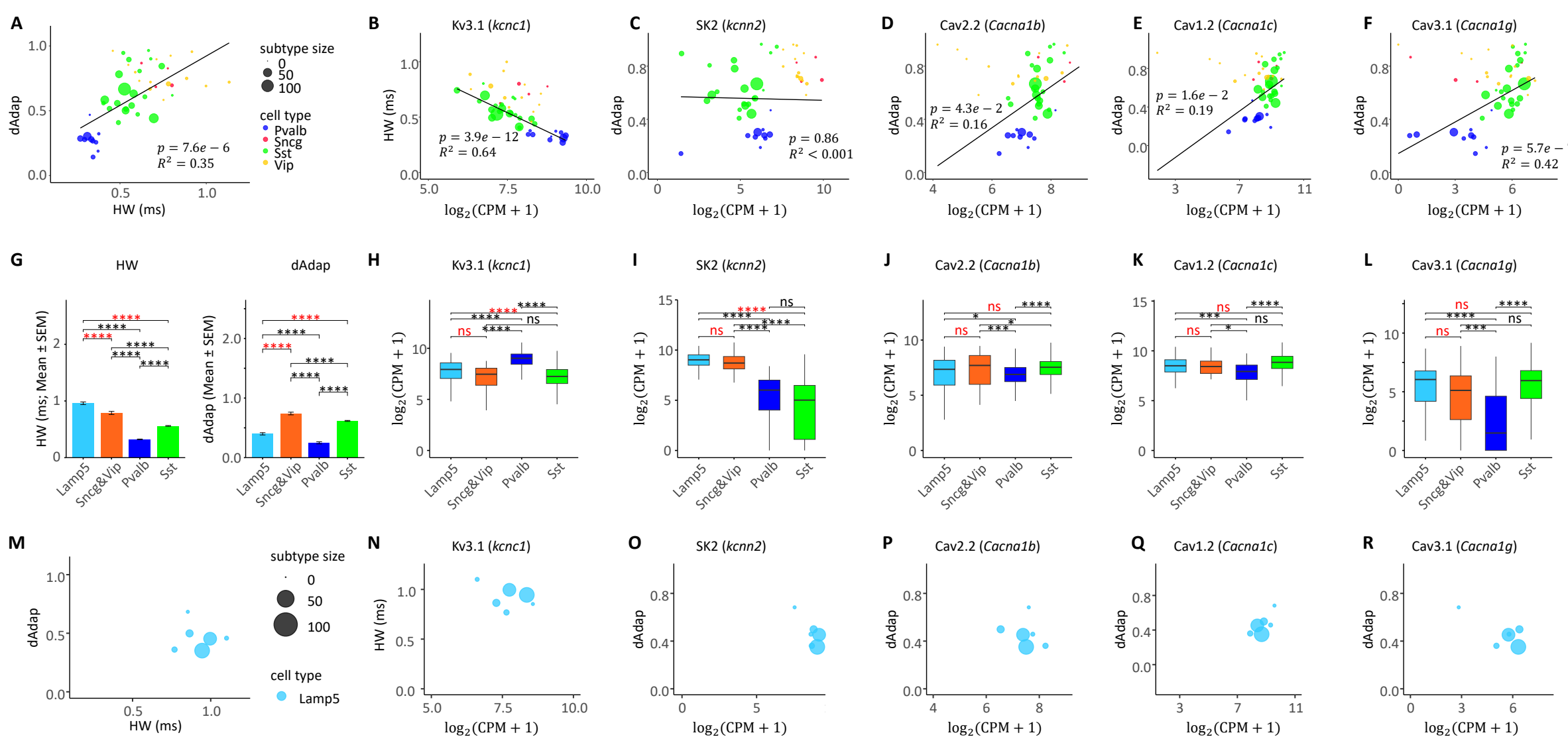

Figure S4: Generalization to VIP, SNCG INs, but not LAMP5 INs. Related to Figure 2. (A-F) Bubble plots that include MGE-derived PV and SST INs, and MGE-derived VIP and SNCG subtypes. (G-L) shows the bar plots and box plots comparing cell types. Since the VIP and SNCG show similar behavior, we grouped them together for simplification. The significance of bar plots is calculated from a two-sided t-test, while the significance of the box plots is calculated from DESeq analysis. (**: $p < 0.01$, ***: $p < 0.001$, ****: $p < 1e-4$). The significances between CGE- and MGE-derived INs are highlighted in red. (H-R) The scatter plots of LAMP5 INs are organized similarly to (A-F). Here, the subtype size is enlarged for clarity.

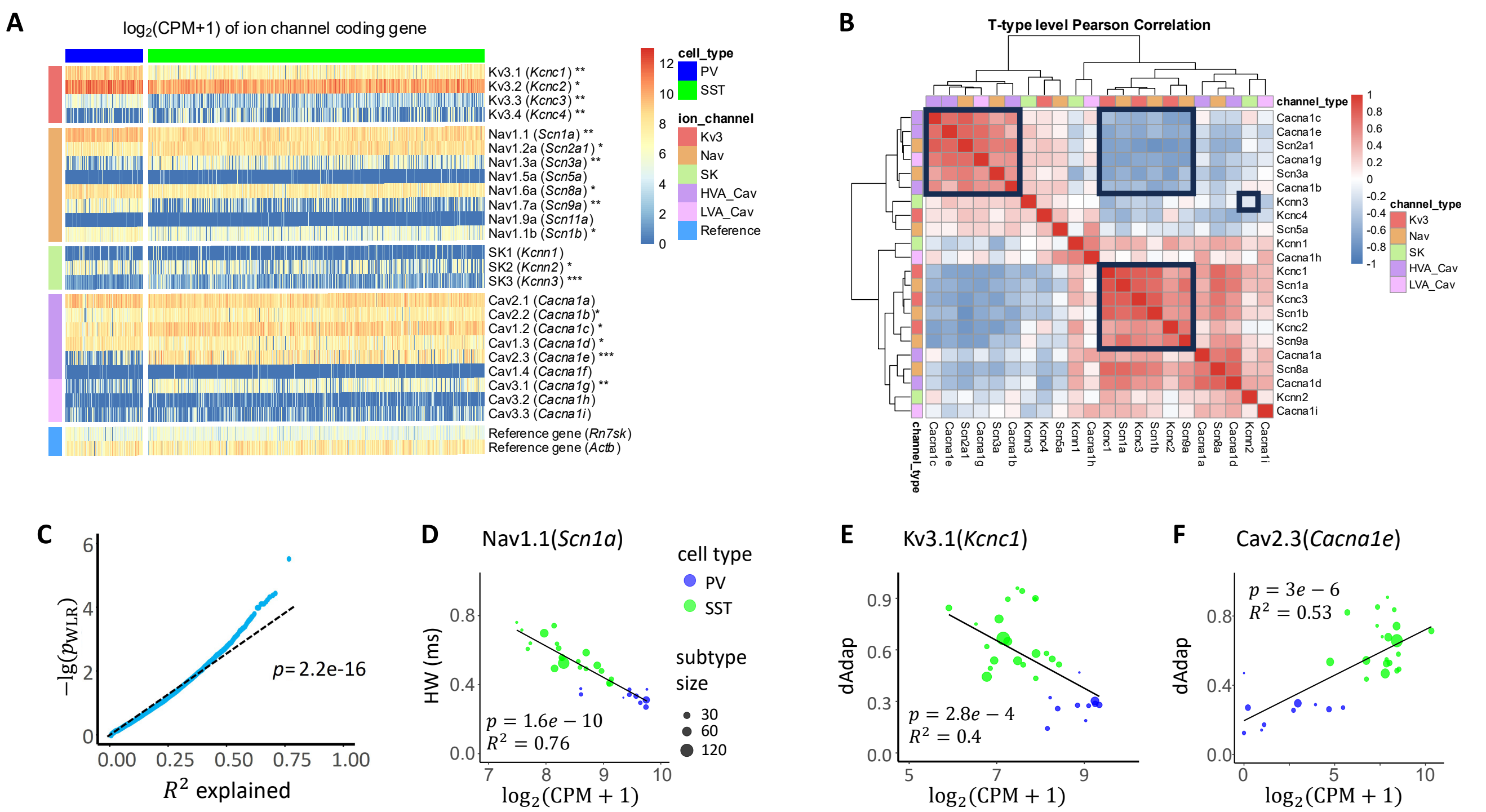

Figure S5: Further analyses of directly related genes. Related to Figure 3. (A) Heatmap of $\log_2(\mathrm{CPM}+1)$ gene expression level at the single-cell level. Genes in rows and cells in columns. Asterisks are used to annotate differential expression significance between V1 PV and SST cells (*: fold change between 0.5 and 1, **: 1 to 2, ***: > 2). In our analysis, genes with $q < 0.05$ and fold change larger than one were counted as DEGs. (B) Person correlation of gene expression level. The boxed regions show the two groups of genes (group 1: *Cacna1c*, *Cacna1e*, *Cacna1g*, *Scn2a1*, *Scn3a*; group 2: *Kcnc1-3, Scn1a, Scn1b, Scn9a*) that are highly correlated within the group and anti-correlated across groups. Also, *Kcnn2* and *Kcnn3* are anti-correlated. (C) R^2 and $-\lg(p_{WLR})$ are linearly correlated. (D) Correlation between HW and *Scn1a*. (E) Correlation between dAdap and *Kcnc1*. (F) Correlation between dAdap and *Cacna1e*.

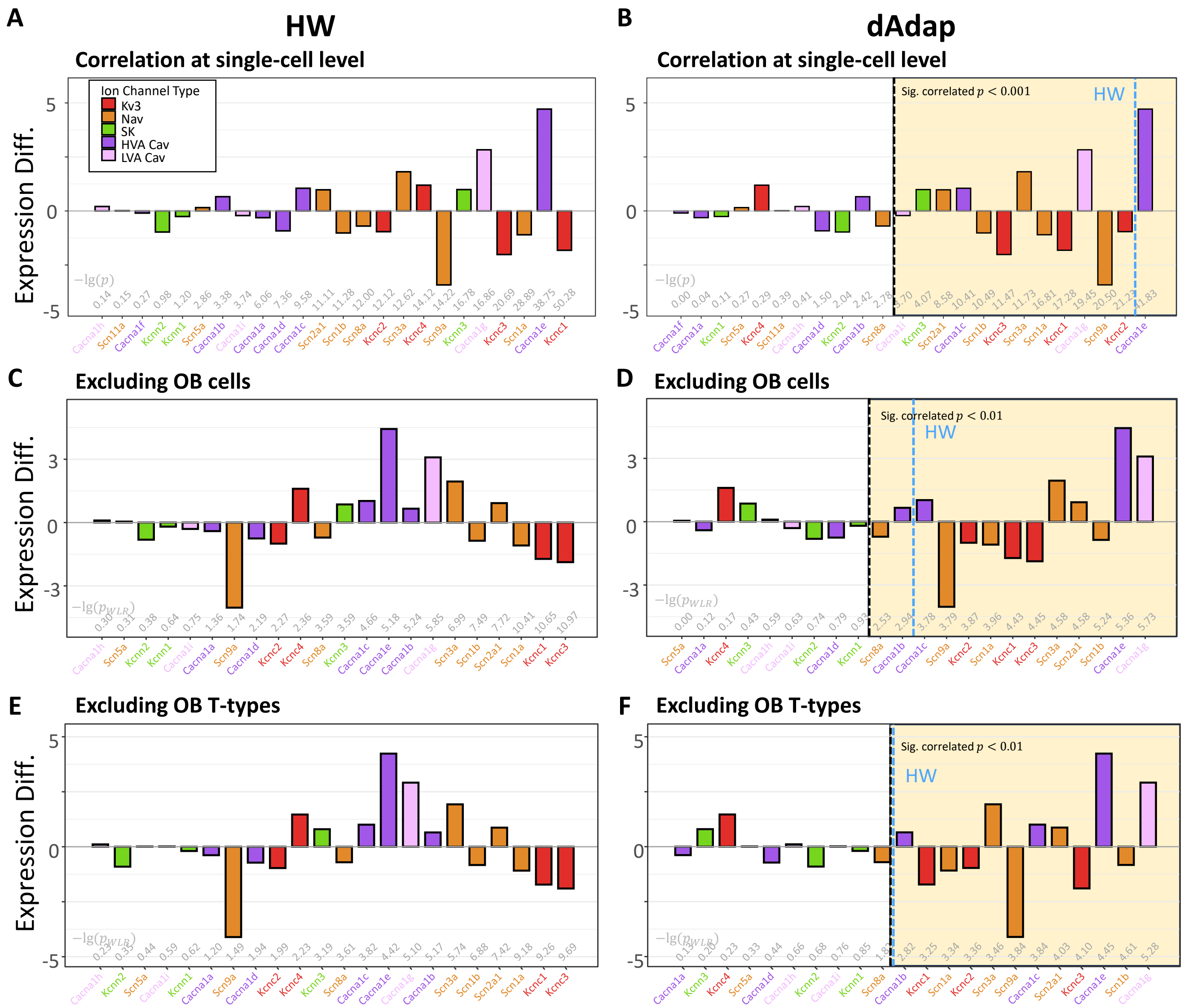

Figure S6: More analysis on the related genes. Related to Figure 3A, B. (A, B) Ranked genes that correlated with HW and dAdap, while the significance is calculated based on the fitting at the single-cell level. The expression difference is measured by the fold change of gene expression. The dashed area indicates the genes significantly correlated with the corresponding e-feature. The Blue dashed line indicates $-\lg(p)$ of HW. (C, D) Ranked genes that correlated with HW and dAdap, while excluding cells with OB features, and the significance is calculated based on the weighted linear regression $-\lg(p^{WLR})$, as in Figure 3. (E, F) Ranked genes, while excluding T-types that have at least one-third of cells showing OB features.

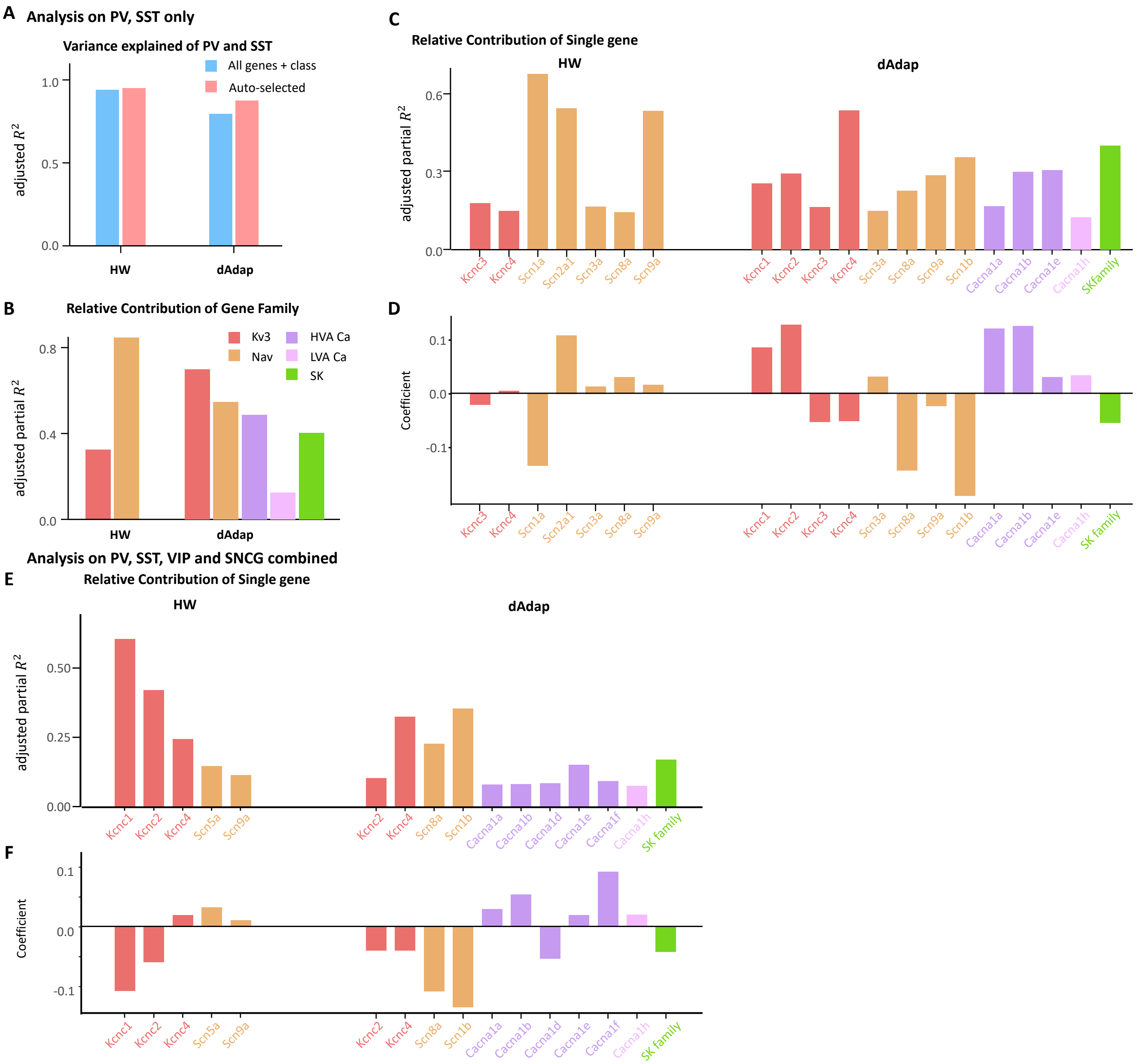

Figure S7: More analysis on the regression model. Related to Figure 3C, D. (A to D) Regression model limited to the PV and SST INs only. (A) Bar plot of adjusted R^2 from multivariate regression models predicting HW or dAdap from candidate genes. Blue bars: models including all related genes and a class-dependent variable (HW: 94.1%, dAdap: 79.5%; see Methods). Pink bars: models based on auto-selected genes (HW: 95.1%, dAdap: 87.6%). (B) Relative contribution of each ion channel family, quantified by adjusted partial R^2. (C) Relative contribution of each gene in the auto-selected model based on the adjusted R^2. Since the subunits encoded by SK-family genes can form a heterotetramer[? ?], we use the summed SK expression values as a single independent variable. (D) The fitted coefficient of each gene in the auto-selected model. Notice that the contribution of the SK family is negative, which implies SK genes contribute negatively to the dAdap, contrasting with the biological understanding. (E, F) Relative contribution and the coefficient of each gene of the auto-selected model for PV, SST, VIP, and SNCG INs while excluding all the OB cells. The analysis on the single cell is less reliable than that at the gene family level, since the large correlation between single genes, as observed in Figure S5B.

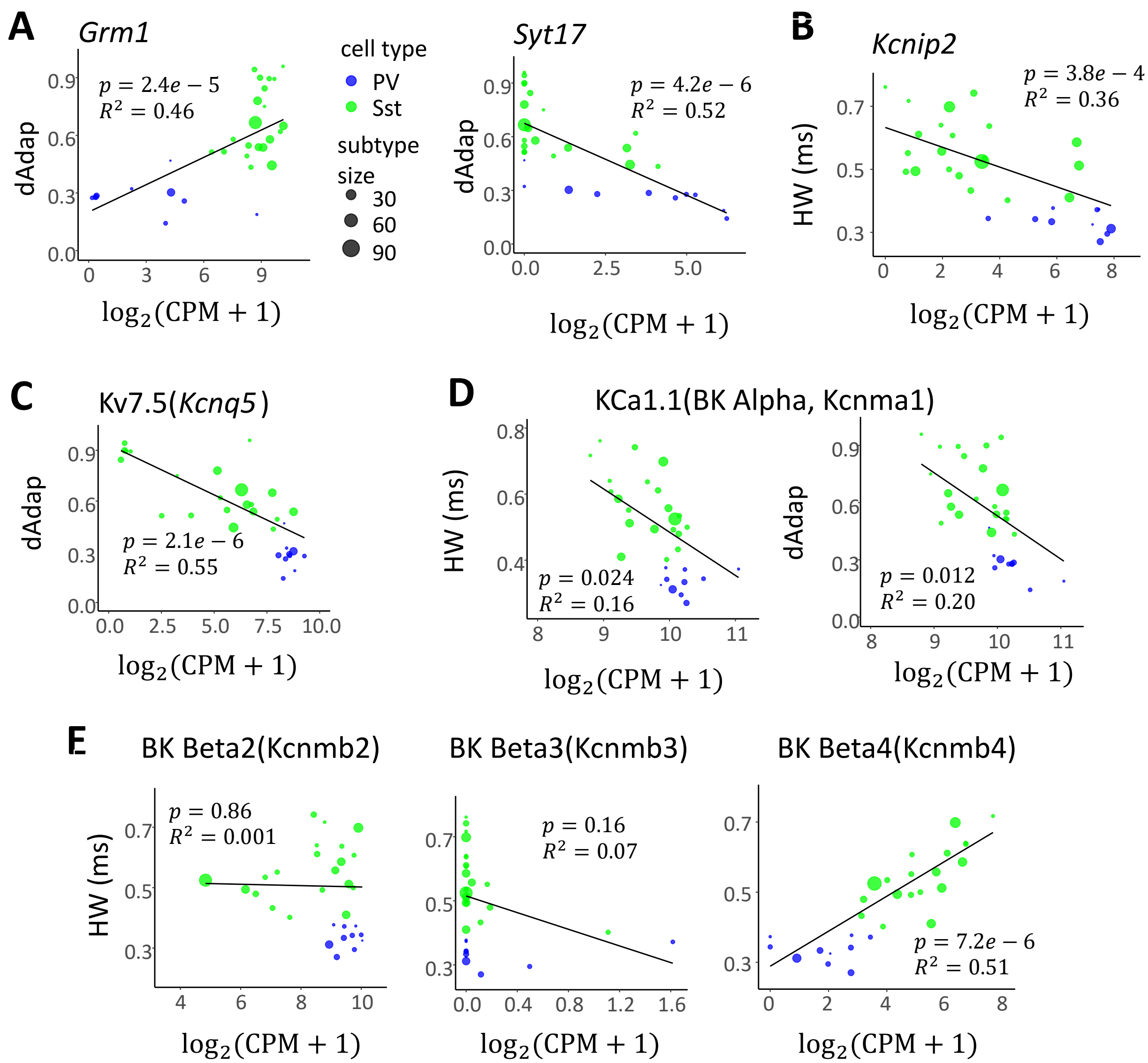

Figure S8: More WLR on example genes. Related to Figure 4. (A) *Grm1* and *Syt17* are highly correlated with dAdap. (B) *Kcnip2* is significantly correlated with HW. (C) *Kcnq5*, encoding voltage-gated potassium channel subunit Kv7.5, is significantly correlated with dAdap. (D) *Kcnma1*, encoding the alpha-unit of big-conductance K^+ (BK) channel, are weakly correlated with HW and dAdap. (E) *Kcnmb2* to *Kcnmb4*, encoding beta-units of the BK channel, do not consistently correlate with HW.

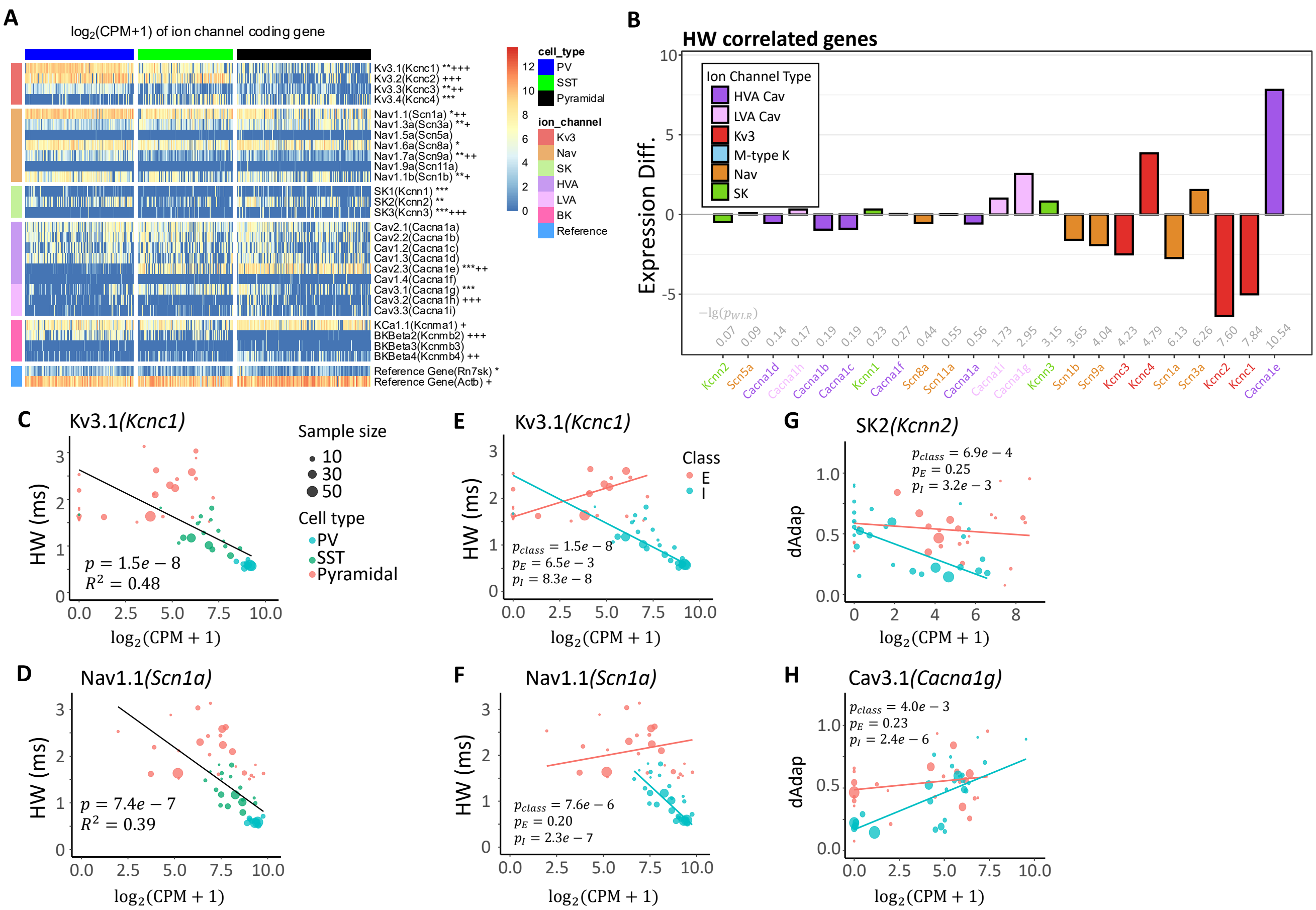

Figure S9: Further analyses on the mouse M1 dataset. Related to Figure 5. (A) Single-cell level heatmap and differential expression analysis. The asterisks(*) and crosses(+) represent the fold change between PV and SST, or INs and pyramidal cells, respectively (*/+: fold change between 0.5 and 1, **/++: 1 to 2, ***/+++: $>$ 2). (B) Ranked genes that correlated with HW. The expression difference is measured by fold change. (C-D) Correlation between HW and *Kcnc1* (C) and *Scn1a* (D) across Pyramidal, SST, and PV cells. (E-H) Class-dependent effect and within-class fitting results of different genes. p_{class} shows the significance of the class-dependent effect; p_E and p_I show the significance of a non-zero slope in the I and E subsets, respectively. E and I subsets are labeled with pink and blue. See Sheet S5.

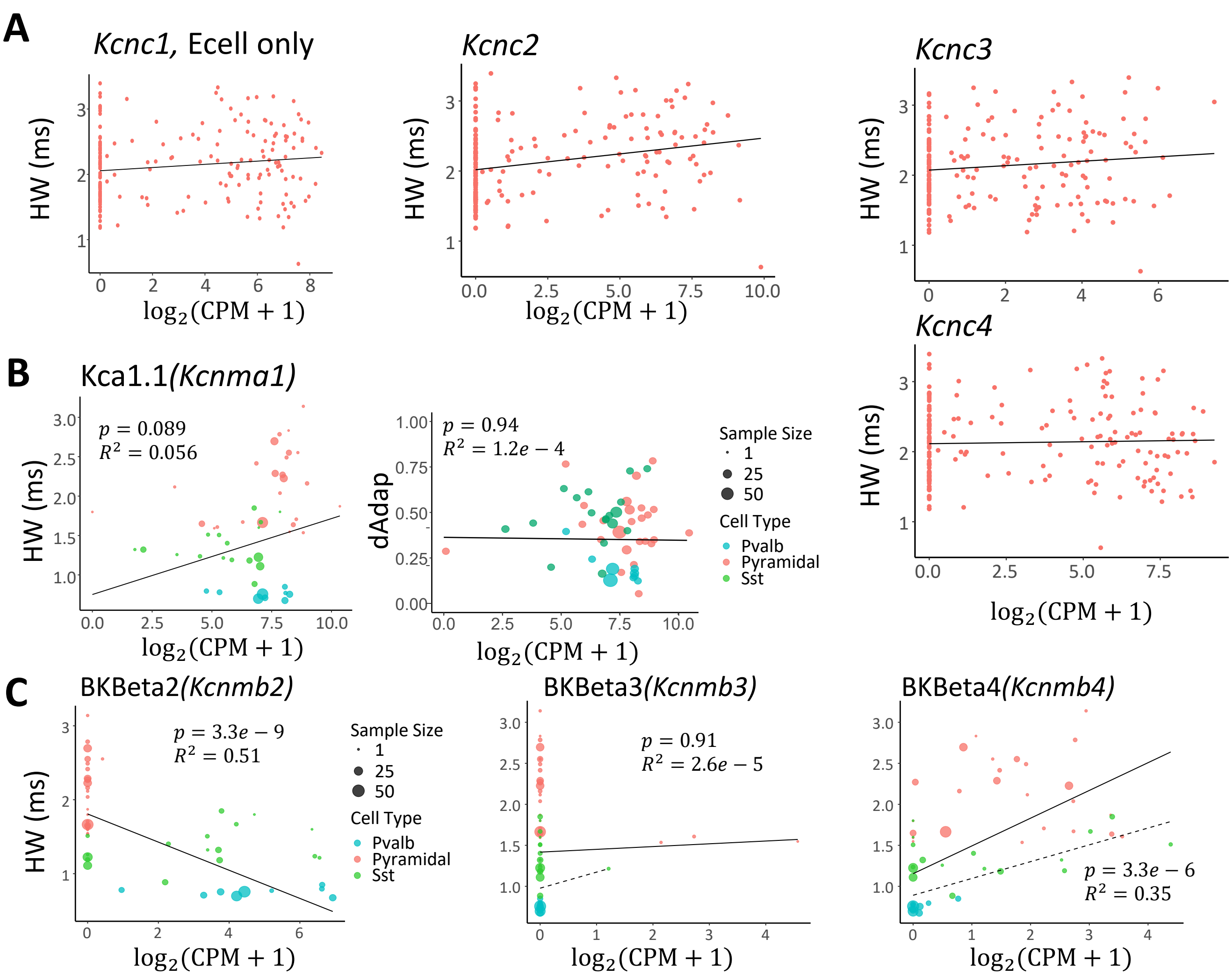

Figure S10: HW differences in M1 pyramidal cells can not be explained by other K channels. Related to Figure 5. (A) correlation between Kv3 encoding genes (*Kcnc1* to *Kcnc4*) and HW at the single-cell level for pyramidal cells. All non-zero slopes are non-significant. (B) Correlation between BK alpha unit *Kcnma1* and HW or dAdap. (C) Correlation between BK beta units *Kcnmb2* to *Kcnmb4* and HW. Based on (B, C), BK encoding genes cannot explain the observed HW difference within pyramidal cells.

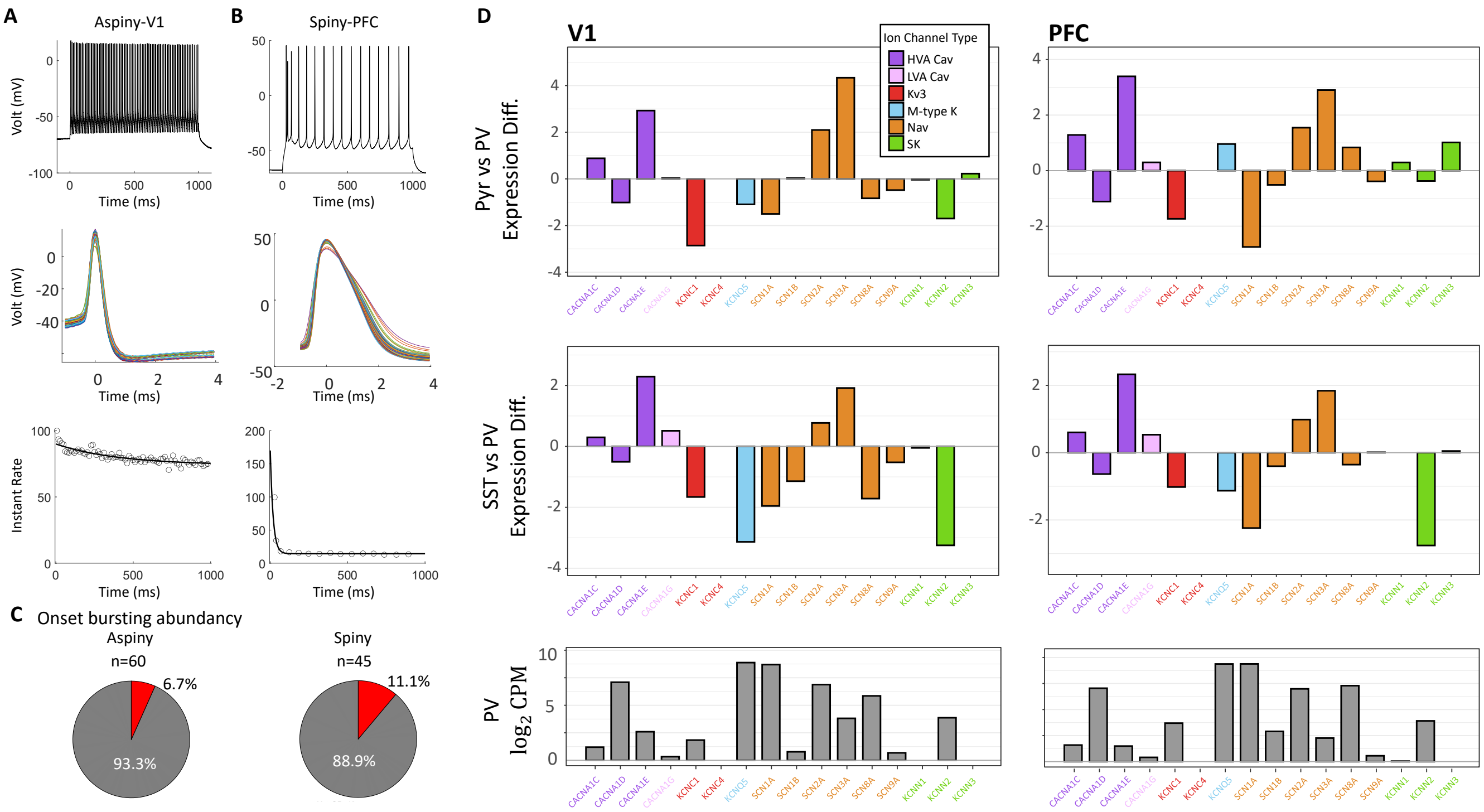

Figure S11: Physiology data analysis of macaque monkeys. Related to Figure 6. (A) Different electrophysiological features from a PFC spiny cell, a putative pyramidal cell. From top to bottom: recording over 1s-long square-pulse current injection; AP waveform; instantaneous firing rate as one over interspike intervals. (B) Same as (A) but from a V1 aspiny cell, a putative interneuron. (C) Abundance of OB features in aspiny cells (left) or spiny cells (right). (D) The expression difference is measured by $\log_2$ fold change of gene expressions in pyramidal (top) and SST cells (middle) compared to that in PV cells at V1 (left) and PFC (right). The mean expressions in PV cells are shown at the bottom. Ion channel families are color-labeled. $N_{\text{SST}}^{\text{V1, PFC}} = \{1.1e3, 8.5e3\}$

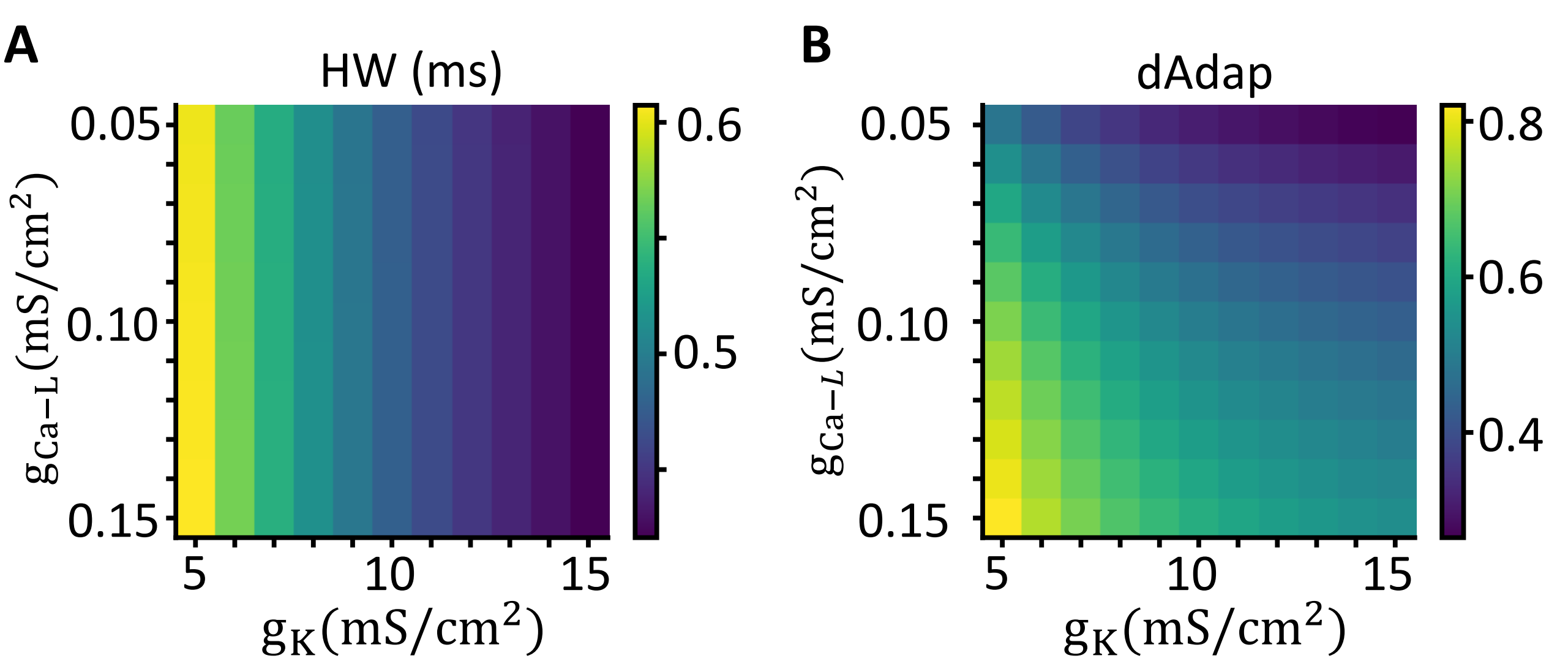

Figure S12: Parameter dependence of HW and dAdap. Related to Figure 7. Effects of varying g_K and g_{Ca-L} on HW (A) and dAdap (B). Reducing g_{Ca-L} selectively decreases dAdap without affecting HW.
